# S100A6 is a critical regulator of hematopoietic stem cells

**DOI:** 10.1038/s41375-020-0901-2

**Published:** 2020-06-19

**Authors:** Tan Hooi Min Grahn, Abhishek Niroula, Ákos Végvári, Leal Oburoglu, Maroulio Pertesi, Sarah Warsi, Fatemeh Safi, Natsumi Miharada, Sandra C. Garcia, Kavitha Siva, Yang Liu, Emma Rörby, Björn Nilsson, Roman A. Zubarev, Stefan Karlsson

**Affiliations:** 1 Division of Molecular Medicine and Gene Therapy, Lund Stem Cell Center, Lund University Hospital, 22184 Lund, Sweden; 2 Hematology and Transfusion Medicine, Department of Laboratory Medicine, Lund University, BMC B13, SE-221 84 Lund, Sweden; 3 Department of Medical Biochemistry and Biophysics, Karolinska Institutet, Solnavägen 9, SE-171 65 Solna, Sweden; 4 Division of Molecular Hematology, Lund Stem Cell Center, Lund University Hospital, 22184 Lund, Sweden; 5 Department of Molecular, Cell and Developmental Biology, Eli and Edythe Broad Stem Cell Research Center, University of California, Los Angeles, CA USA; 6 Experimental Hematology Unit, Department of Clinical and Experimental Medicine, Linköping University, Linköping, Sweden

**Keywords:** Haematopoietic stem cells, Cell signalling

## Abstract

The fate options of hematopoietic stem cells (HSCs) include self-renewal, differentiation, migration, and apoptosis. HSCs self-renewal divisions in stem cells are required for rapid regeneration during tissue damage and stress, but how precisely intracellular calcium signals are regulated to maintain fate options in normal hematopoiesis is unclear. S100A6 knockout (KO) HSCs have reduced total cell numbers in the HSC compartment, decreased myeloid output, and increased apoptotic HSC numbers in steady state. S100A6KO HSCs had impaired self-renewal and regenerative capacity, not responding to 5-Fluorouracil. Our transcriptomic and proteomic profiling suggested that S100A6 is a critical HSC regulator. Intriguingly, S100A6KO HSCs showed decreased levels of phosphorylated Akt (p-Akt) and Hsp90, with an impairment of mitochondrial respiratory capacity and a reduction of mitochondrial calcium levels. We showed that S100A6 regulates intracellular and mitochondria calcium buffering of HSC upon cytokine stimulation and have demonstrated that Akt activator SC79 reverts the levels of intracellular and mitochondrial calcium in HSC. Hematopoietic colony-forming activity and the Hsp90 activity of S100A6KO are restored through activation of the Akt pathway. We show that p-Akt is the prime downstream mechanism of S100A6 in the regulation of HSC self-renewal by specifically governing mitochondrial metabolic function and Hsp90 protein quality.

## Introduction

Hematopoiesis is defined by the generation of the cellular components of the blood system. Hematopoietic stem cells (HSCs) are capable of regenerating different blood cell types upon demand through a variety of signaling and differentiation pathways [1], and this rare population offers promising opportunities for stem-cell-based therapies in combatting hematological disorders [2].

Mitochondria are involved in hematopoiesis and HSC fate decisions [3, 4]. HSCs exhibit lower baseline energy production and lower maximal respiration than progenitor cells despite higher levels of mitochondrial content in HSCs [5, 6]. Mitochondria are proposed to function as an important organelle to determine HSC fate decisions and disintegration of mitochondria leads to severe effects in cellular function [4, 7, 8]. Conditional knockout (KO) of the gene *Uqcrfs1*, an essential subunit of the mitochondrial complex III has been reported to lead to severe defects in stem cell properties [4]. Similarly, conditional deletion of the *SdhD* gene, a subunit of the mitochondrial complex II is essential for HSC survival and maintenance [7].

Intracellular Ca^2+^/cytosolic Ca^2+^ (Ca_i_^2+^) is an important secondary messenger that regulates several intracellular pathways. The SCF/c-kit HSC pathway stimulates the participation of Ca_i_^2+^ in hematopoiesis [9]. A previous report has shown that calmodulin-dependent protein kinase IV (Camk4) is involved in the maintenance of HSCs [10]. Calmodulin is a Ca^2+^-signaling protein and has the conserved calcium-binding motif named the EF hand [11]. Although the S100 protein family has conserved functional domain of two distinct EF-hands like calmodulin, S100 proteins have tissue-specific intra- and extracellular functions [12]. S100A6 (calcyclin) is a member of the EF-hand family of calcium-binding proteins and is found increased when quiescent cells are stimulated to proliferate [13]. Recently, S100A6 was found expressed in neural stem cells and it was also secreted from mesenchymal stem cells [14], we therefore hypothesize that S100A6 is a potential regulator of HSC self-renewal. S100A6 expression is known to be upregulated in leukemia with poor prognosis and it exerts antiapoptotic effects in mixed-lineage leukemia (MLL)/AF4-positive leukemia cells [15–17]. However, to develop targeting of S100A6 for treatment of myeloid leukemia, it is essential to understand how loss of S100A6 affects normal blood development. S100A6 interacts with heat shock proteins Hsp70/Hsp90 complexes [18], but the mechanistic pathway of S100A6 and Hsp90 regulation in HSC is not known. The SDF-1/CXCR4 axis is an Akt activator [19] and stem cell factor (SCF) binds to the c-kit receptor activated Akt signaling to regulate cellular survival [20]. We are interested to explore if S100A6 mediates the Akt and Hsp90 survival pathways in a calcium-dependent manner upon SDF-1 or SCF stimulation. A recent study demonstrated that the combination of cytokine stimulations and Ca^2+^-mitochondria pathway is crucial for HSC division during stress [21], but its regulation is unclear in normal hematopoiesis and the role of S100A6-Ca_i_^2+^ in HSC maintenance is unknown. Therefore it is important to explore how S100A6 regulates HSCs fate options by the intracellular and mitochondrial Ca^2+^ level under normal physiological condition.

In this work, we have demonstrated that S100A6 is a critical regulator of HSCs self-renewal by inducing high engraftment activity in HSC serial transplantations. Our results demonstrate for the first time that S100A6 furnish the antiapoptotic effects in murine HSC, which are supported by several investigations in other systems [15–17]. Our findings show that the calcium-dependent S100A6 governs the Akt activation pathway and this includes the regulation of mitochondrial calcium levels, respiratory metabolism, Hsp90 protein, and HSC survival.

## Materials and methods

### The mouse model

To study the in vivo function of S100A6 during maintenance of adult hematopoiesis, mice on C57BL/6 background homozygous for the conditional *S100a6*^*flox*^ allele were crossed with mice harboring the *Vav-Cre* transgene (Fig. S1a) [22] to produce *Vav-Cre;S100a6*^*flox/flox*^ mice (mutant) and their Vav-negative littermate controls (control). To obtain *Vav-Cre;S100a6*^*ΔΔ*^(KO), *Vav-Cre;S100a6*^*flox/flox*^ were bred with *Vav-Cre;S100a6*^*flox/flox*^ *Vav-Cre* for several generations. *S100a6*^*ΔΔ*^KO mice were studied in comparison with control mice to determine any possible abnormalities in hematopoietic cells in the S100A6KO. Females and males mice aged 8–16 weeks old were used in all experiments, randomized, with matched littermate controls. Mice were housed and bred in ventilated cages in the BMC animal facility. The regional Animal Ethical Committee in Lund approved all animal experiments.

### RNA sequencing

Raw RNA sequencing reads have been submitted to Sequence Read Archive (accession: PRJNA578124). The data will be accessible on publication of the manuscript (https://www.ncbi.nlm.nih.gov/bioproject/PRJNA578124).

### Protein identification

Proteins with *p* values above the confidence level of 0.05 were submitted to STRING search (https://string-db.org), identifying biological functions and related pathways. The mass spectrometry proteomics data have been deposited to the ProteomeXchange Consortium via the PRIDE partner repository with the dataset identifier PXD015854 (http://www.ebi.ac.uk/pride).

## Results

### S100A6 is expressed in long-term hematopoietic stem cells (LT-HSC) and regulates stem cells specific transcripts

**Fig. 1:**
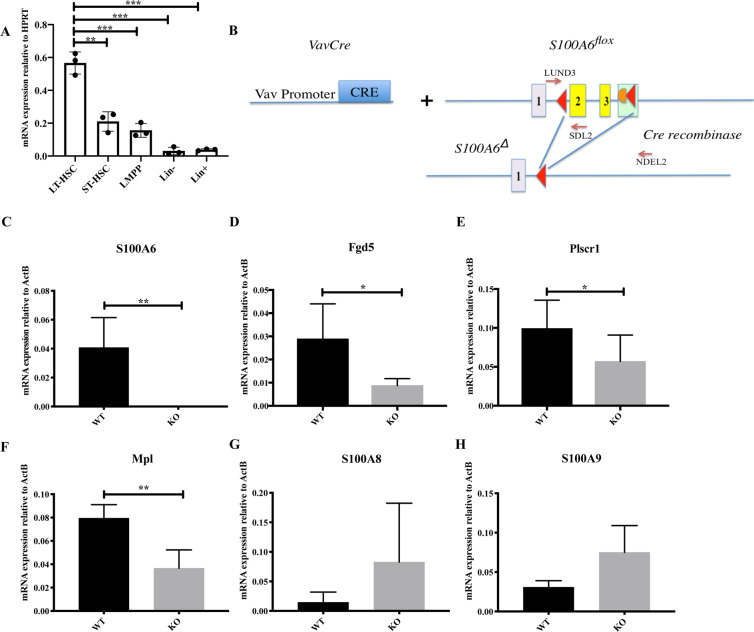
S100A6 is highly expressed in LT-HSCs and several stem-cell-specific transcripts are decreased in the absence of S100A6. **a** Quantitative real-time PCR (qRT-PCR) analysis of *S100a6* expression in long-term HSC (LT-HSCs; (lineage^−^Scal-1^+^ c-Kit^+^) LSK CD34^−^Flt3^−^), short-term HSC (ST-HSC; LSK CD34^+^Flt3^−^), lymphoid-primed multipotent progenitors (LMPP; LSK CD34^+^Flt3^+^), lineage^−^, and lineage^+^ cells. Each value is normalized to HPRT expression and mean ± SD of triplicates is shown (*n* = 3; *p < 0.05; **p < 0.001; ***p < 0.0001; analyzed by an unpaired two-sided *t*-test). **b** Schematic representation of *Vav-Cre*-mediated conversion of the *S100a6*^*flox*^ allele into the *S100a6*^*Δ*^ allele by deletion of DNA between the two loxP sites in the *S100a6*^*flox*^ locus. This includes the entire *S100a6* exons 2 and 3 (yellow). Exons 1–3 are indicated. Red triangles are loxP sites. Orange semisphere is FRT site from excised neo cassette (green rectangular). *Cre recombinase* cleaved at loxP sites. **c** *S100a6* mRNA expression in BM HSCs (CD150^+^, CD48^−^, Flt3^−^, CD34^−^) (*n* = 4). mRNA levels of *Fgd5* (*n* = 4) (**d**), *Plscr1* (*n* = 8) (**e**), *Mpl* (*n* = 4) (**f**), *S100A8* (*n* = 4) (**g**), *S100A9* (*n* = 3) (**h**), assessed by qRT-PCR on LT-HSCs (CD150^+^CD48^−^CD34^−^Flt^−^). Results are the mean ± SD of triplicates. Each value is normalized to *ActB* expression (*p < 0.05; **p < 0.001; analyzed by an unpaired two-sided *t*-test).

To assess the expression pattern of S100A6, we sorted HSCs from mouse bone marrow using cell surface markers CD34 and Flt3 within the LSK (lineage^−^Sca-1^+^ c-Kit^+^) compartment. *S100a6* transcripts were abundantly expressed in LT-HSC compared with other more differentiated populations (Fig. 1a).

To investigate the functional activity of S100A6 in HSCs and to determine whether S100A6 is a critical regulator of HSCs, we created a S100A6 conditional mouse KO (S100A6KO) in the hematopoietic system (Figs. 1b and  S1a). Using a web interface, we visualized *S100a6* gene expression in HSPCs at single-cell resolution. This interface displays single-cell RNA-seq data from 1656 single index-sorted HSPCs. *S100a6* is highly expressed in cluster 1 (blue), which consists in majority of LT-HSCs (79%) (Fig. S1b) [23].

As expected quantitative real-time PCR (qRT-PCR) analysis neither detect *S100a6* expression in the S100A6KO cells in bone marrow cells sorted with surface markers (CD150^+^, CD48^−^, Flt3^−^, and CD34^−^) (internal control in each experiment) (Fig. 1c), nor was the *S100a6* mRNA detected in the c-kit-enriched bone marrow cells (Fig. S1c). Expression levels of several HSC transcripts (*Fgd5*, phospholipid scramblase 1 (*Plscr1*), and *Mpl*) were downregulated in S100A6-deficient HSCs (Fig. 1d–f). S100 family members *S100a8* and *S100a9* showed no significant difference (Fig. 1g, h). Transcripts of seven S100 family members (*S100a1*, *S100a4*, *S100a8*, *S100a9*, *S100a10*, *S100a11*, and *S100a13*) were found in our RNA-seq gene list but showed no significant difference between the WT and the KO (data not shown). Notably, S100A6 null mice were born at normal Mendelian ratios and matured to adulthood without any obvious defects (data not shown).

Our finding indicates that S100A6 is specifically involved in HSC regulation without obtaining compensatory functions from the S100 family members.

### S100A6KO mice show reduced LT-HSC and multipotent progenitor (MPP) populations with increased apoptotic cells in the LT-HSC compartment

**Fig. 2:**
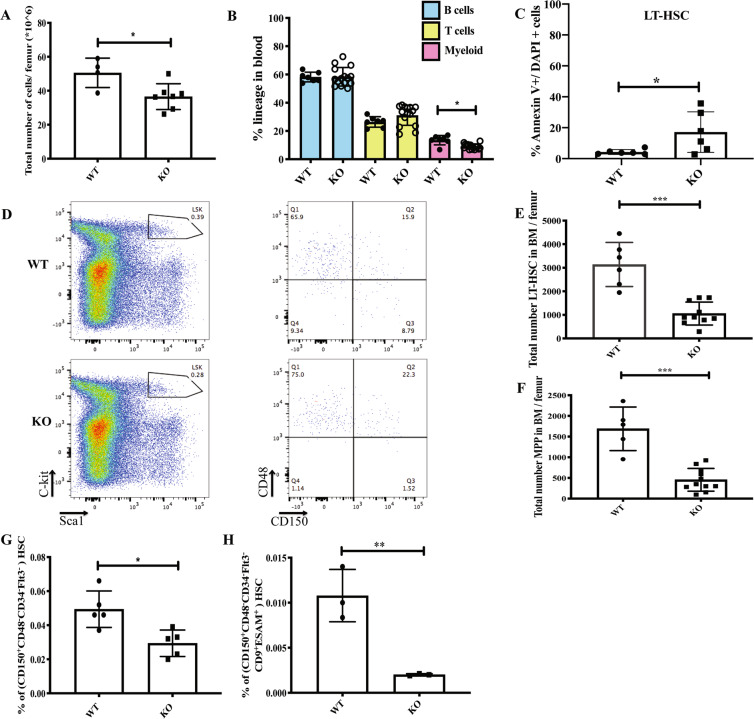
S100A6-deficient mice display reduced steady-state hematopoietic stem and progenitor cells. **a** Total number of whole bone marrow cells is reduced in S100A6KO mice. **b** Lineage distribution in steady-state peripheral blood. **c** S100A6KO HSCs have significant higher levels of apoptotic cells. **d** Schematic gating of LSK, CD150, CD48 HSPC compartment, in steady-state whole bone marrow cells. Representative FACS plots of LT-HSC (CD150^+^CD48^−^) and multipotent progenitor (MPP, CD150^−^CD48^−^). S100A6KO had a robust reduction in the LT-HSC and MPP compartments compared with WT. **e**, **f** Histogram summarized total cells number within the LT-HSC and MPP in bone marrow. **g** C-kit-enriched FACS sorted bone marrow cells (LSK CD150^+^CD48^−^CD34^−^Flt3^−^). **h** More stringent gating of FACS sorted c-kit-enriched bone marrow cells (LSK CD150^+^CD48^−^ CD34^−^Flt3^−^ESAM^+^CD9^Hi^) (*p < 0.05; **p < 0.001; ***p < 0.0001; all data were analyzed by unpaired two-sided *t*-test and Mann–Whitney *U* test). All individual dots represent randomized biological replicates with matched littermates and experiments were repeated three times.

To examine the effect of the S100A6 conditional deletion in steady-state hematopoiesis, we performed FACS analyses on murine blood and BM. Total cellularity decreased significantly in S100A6-deficient mice (Fig. 2a). In addition, steady-state S100A6KO had a lower myeloid lineage output in peripheral blood (Fig. 2b). Given the low total cellularity (Fig. 2a), we investigated the apoptotic status of steady-state S100A6KO by analyzing Annexin V and DAPI expression levels (Fig. S2a) in the LSKCD150^+^CD48^−^ compartment. We found that S100A6KO mice had increased Annexin V^+^/DAPI^+^ expression in the LT-HSCs (Fig. 2c). Immunophenotypic analyses in BM based on surface markers (LSKCD150^+^CD48^−^) for S100A6WT (Fig. 2d, upper row) and S100A6KO (Fig. 2d, lower row) were performed. S100A6KO mice exhibited significantly lower number of total cells within the LT-HSC (CD150^+^CD48^−^) (Fig. 2e) and MPP (CD150^−^CD48^−^) (Fig. 2f). We found a significant reduction of LT-HSC from the sorted source of LT-HSC (LSKCD150^+^CD48^−^CD34^−^Flt3^−^) after c-kit enrichment in S100A6KO (Figs. 2g and S2b). Previous studies have reported that CD9 and ESAM are HSC markers that can capture pure murine HSCs [24, 25]. We further isolated purified LT-HSCs using a more stringent gating strategy (LSKCD150^+^CD48^−^CD34^−^Flt3^−^CD9^Hi^ESAM^+^) and identified a similar reduction in this LT-HSCs fraction (Fig. 2h).

Our data demonstrate that the S100A6 deletion perturbs the HSPC compartment, significantly reducing LT-HSC and MPP numbers and myeloid output with increased number of apoptotic cells in the LT-HSC compartment.

### S100A6KO HSCs have reduced cytosolic calcium signaling, reduced Akt signaling, and reduced mitochondrial aerobic activity

**Fig. 3:**
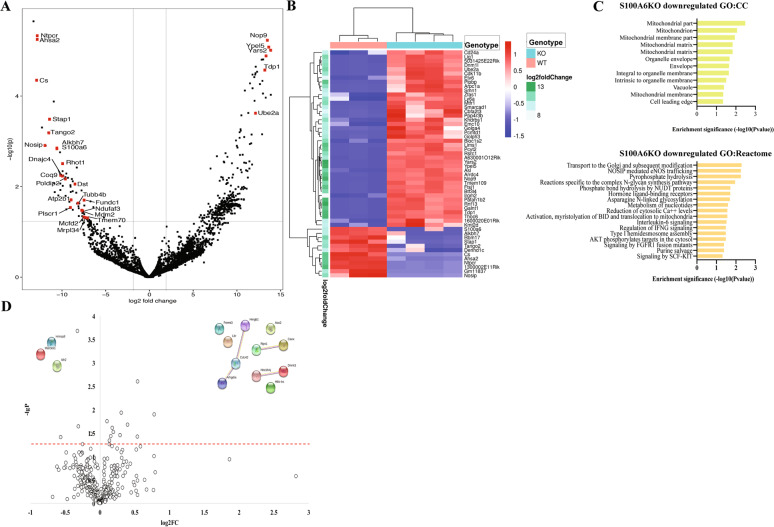
Gene expression analysis shows that S100A6 null HSCs have reduced transcripts of genes that regulate intracellular calcium, Akt signaling, and mitochondria aerobic activity; differential protein expression between S100A6WT and KO HSCs indicates reduction of Hsp90 protein. **a** Volcano plot of RNA-seq showing log fold change and negative logarithm of *p* values between WT and S100A6-deficient LT-HSC (CD150^+^CD48^−^CD34^−^Flt^−^). **b** Heatmap summarizing expression of 54 genes exhibiting differential expression between WT (pinkish-orange) and KO (light blue) at steady state. Samples are in the columns, genes in the rows, and the standardized expression levels are depicted by the color gradient: upregulated genes in red, downregulated genes in blue, adjusted *p* value < 0.2. **c** (above) Gene ontology enrichment analysis of cellular components for downregulated genes in S100A6-deficient samples. (Below) Gene ontology enrichment analysis of Reactome for downregulated genes in KO samples (accessed NetworkAnalyst 3.0). **d** Volcano plot of protein relative abundances between S100A6WT and KO at steady state. The *X*-axis shows the fold change in logarithmic scale. The *Y*-axis shows the log of the *p* value. Horizontal dashed line corresponds to *p* value = 0.05.

To elucidate the molecular consequences of S100A6 loss in HSCs, we executed a transcriptome-wide expression analysis using mRNA sequencing of purified LT-HSCs (LSKCD150^+^CD48^−^CD34^−^Flt3^−^) from both S100A6WT and KO. The differential expression analysis revealed 448 differentially expressed genes (380 upregulated and 68 downregulated, *p* < 0.05). S100A6 was ranked as the seventh most downregulated gene (*p* value: 0.001) (Figs. 3a and  S3a). We presented the first 54 genes with adjusted *p* < 0.2 (42 upregulated; 12 downregulated in the KO) on a heatmap. Our heatmap showed that the genes with similar expression patterns are clustered together (Fig. 3b). We performed gene-set network in terms of their associated biological process, 12 downregulated genes (Fig. 3b) were enriched in aerobic respirations (Fig. S3b), while 42 upregulated genes (Fig. 3b) were related to intrinsic apoptotic signaling pathway (Fig. S3c).

In order to outline the most important processes of 448 differential expressed genes for the cellular pathways, we undertook a gene ontology enrichment analysis of the 68 downregulated gene set, and we processed these genes in terms of their associated cellular component. The top-ranked cellular component was “mitochondrial part,” and includes the genes *Cs*, *Tmem70*, *Mrpl34*, *Fundc1*, *Ndufaf3*, *Atp5s*, *Poldip2*, and *Rhot1* (Fig. 3c, above). Besides, we mapped these 68 downregulated essential genes to the Reactome described in gene ontology [26], and we found that the top-ranked biological processes that were significantly enriched in Reactome and these include the reduction of cytosolic Ca^++^ levels, signaling by SCF-Kit, and Akt phosphorylates targets in the cytosol (Fig. 3c, below). We also carried out gene set enrichment analysis (GSEA) and downregulation gene sets in the S100A6KO were related to mitochondria respiration and the electron transport chain, but upregulation gene sets were linked to oxidative stress (Fig. S3d). Our transcriptome data suggest that S100A6KO HSCs exhibited metabolic oxidative stress and a deficit in mitochondria function.

As an approach to identify downregulated proteins during S100A6-Ca_i_^2+^ signaling, we used proteomics. We analyzed LT-HSCs (LSKCD150^+^CD48^−^CD34^−^Flt3^−^) applying proteomics. A volcano plot was generated using a *p* < 0.05 by Student’s *t* test. The statistical analyses were performed for comparison between S100A6WT and S100A6KO. One of the most downregulated proteins in S100A6KO LT-HSCs was mitochondrial isocitrate dehydrogenase (IDH2) (Fig. 3d). Intact mitochondria functions depend on a regulated and adaptive protein quality control environment, proper folding of proteins, and efficient elimination of toxic protein aggregates [27]. Our functional enrichment of proteins in the constructed interaction network was carried out online in the STRING database (Fig. S3e). The most interesting enriched GO category in our data was the HSP90 protein pathway showing a downregulation in S100A6KO (Table 1, above), while apoptotic execution phase proteins were upregulated in the KO (Table 1, below), which is in line with our observation from both of increased levels of apoptotic cells in LT-HSC (Fig. 2c) and transcriptomic data (Fig. S3c).

**Table 1:** Enriched GO category derived from STRING database.

	Description	Count in gene set	False discovery rate
S100A6 downregulated protein			
PFAM protein domains			
Domain			
PF00076	RNA recognition motif. (a.k.a. RRM, RBD, or RNP domain)	18 of 228	1.14e−10
PF01423	LSM domain	6 of 21	4.77e−06
PF00118	TCP-1/cpn60 chaperonin family	5 of 15	2.36e−05
PF00183	Hsp90 protein	3 of 4	0.00078
PF10417	C-terminal domain of 1-Cys peroxiredoxin	3 of 6	0.015
			(more…)
S100A6 upregulated protein			
Reactome pathways			
Pathway			
MMU-75153	Apoptotic execution phase	11 of 48	1.18e−10
MMU-109581	Apoptosis	12 of 93	2.02e−09
MMU-211227	Activation of DNA fragmentation factor	7 of 13	5.58e−09
MMU-2559584	Formation of senescence-associated heterochromatin foci (SAHF)	7 of 17	1.62e−08
MMU-2559586	DNA damage/telomere stress induced senescence	9 of 51	1.90e−08
			(more…)

(above) Changed proteins were mapped onto PFAM protein domain according to gene ontology classification system. (below) Changed proteins were mapped onto Reactome pathway according to gene ontology classification system.

Together, both GSEA and data obtained from NetworkAnalyst indicate that S100A6 governs Ca_i_^2+^ signaling, Akt pathway, and mitochondrial aerobic activity. Proteomic data demonstrate that Hsp90 protein appears to be important in S100A6 regulated HSC.

### S100A6KO HSC regulates repopulation of LT-HSCs through the Akt activation pathway

**Fig. 4:**
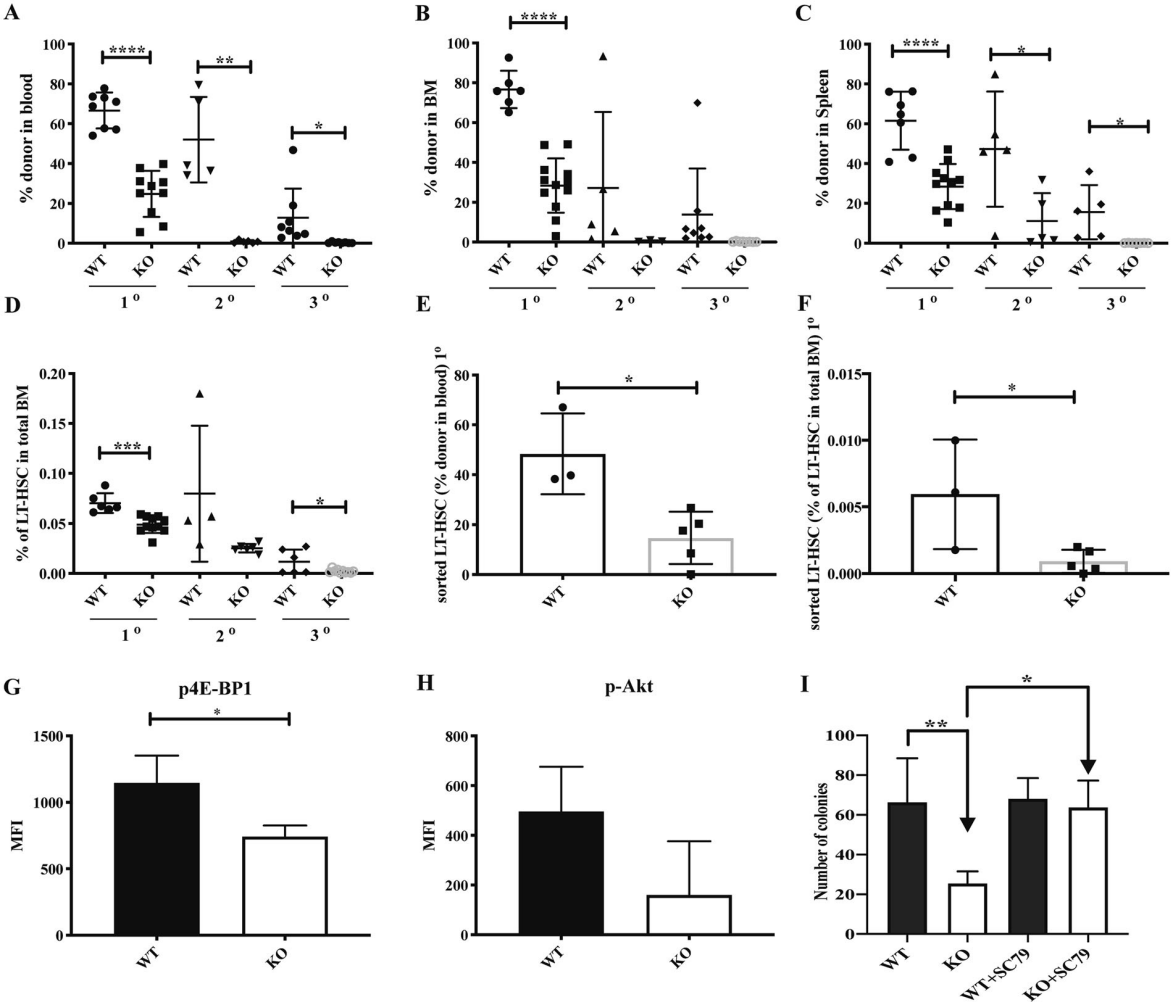
S100A6KO have impaired reconstitution ability of HSCs after transplantation and a decrease in progenitor cell activity, but Akt phosphorylation activator restores the numbers of colonies in vitro. **a**–**c** Whole bone marrow competitive transplantation assays (blood, bone marrow, and spleen at 16 weeks; primary (1°) (WT *n* = 8; KO *n* = 10); secondary (2°) (WT *n* = 5; KO *n* = 6); tertiary (3°) transplantation (WT *n* = 8; KO *n* = 8)). **d** Frequencies of LT-HSCs (LSK CD150^+^CD48^−^) in 1°–3° recipients 16 weeks after whole bone marrow competitive transplants (*n* = similar as (**a**–**c**)). **e** Reduced reconstitution of 50 sorted LT-HSCs in blood determined by CD45.1/45.2 at 16 weeks end point (WT *n* = 3; KO *n* = 5). **f** Frequencies of LT-HSCs (LSK CD150^+^CD48^−^CD34^−^Flt3^−^ESAM^+^CD9^Hi^) in primary recipients 16 weeks end point after intravenous injection of 50 sorted LT-HSCs transplant (WT *n* = 3; KO *n* = 5) (*p < 0.05; **p < 0.001; *** or ****p < 0.0001; all data were examined by unpaired two-sided *t*-test and Mann–Whitney *U* test). **g** Intracellular staining of phosphorylated 4E-BP1 (Thr37/46) in S100A6WT and KO HSCs (CD150^+^CD48^−^CD34^−^Flt^−^) (*n* = 3; *p < 0.05, analyzed by unpaired two-sided *t-*test). **h** Intracellular staining of phosphorylated Akt (Ser473) in HSCs (*n* = 3). **i** S100A6KO bone marrow cells exhibited reduced colony formation capacity in colony-forming unit (CFU) assays. Akt Activator, SC79, 2–8 μg/ml (Abcam) reverted colony-forming capacity in the KO (WT *n* = 4; KO *n* = 6; WT + SC79 *n* = 3; KO + SC79 *n* = 3). **p < 0.001, analyzed by Mann–Whitney *U* test comparing WT with KO. All data above represent mean values from three independent experiments ± SD.

To investigate the functionality of S100A6KO LT-HSCs, a series of competitive transplantations and transplantation of purified LT-HSC were performed. Following competitive whole BM transplantation into irradiated recipient mice, S100A6KO cells competed poorly in comparison to WT littermates and had a significant reduction in reconstitution of blood, bone marrow, and spleen at 16 weeks (Fig. 4a–c). Importantly, the frequency of LT-HSCs (LSKCD150^+^CD48^−^) decreased in S100A6KO HSPC compartments (Fig. 4d). In order to assess whether the repopulation defect of whole BM in S100A6 null cells was specifically caused by an impaired function of LT-HSC, we purified 50 LT-HSC (LSKCD150^+^CD48^−^CD34^−^Flt3^−^ESAM^+^CD9^Hi^) by FACS and the cells were transplanted into lethally irradiated recipients. We observed a robust reduction of regenerative capacity with a reduced reconstitution in blood (Fig. 4e) and failure to repopulate HSPC compartments following primary transplantation of KO but not of WT LT-HSC (Fig. 4f). Despite the impaired reconstitution of blood and LT-HSCs from S100A6KO sorted cells, the recipient mice have normal lineage distribution in blood (Fig. S4a), bone marrow (Fig. S4b), and spleen after 16 weeks (Fig. S4c).

In order to understand the mechanism of S100A6 in relation to Akt signaling in HSCs, we analyzed phosphorylation of key components in these signaling pathways. Intracellular staining analyses of phosphorylated proteins using FACS showed a significant reduction of p4E-BP1, and a decrease in phosphorylated Akt (p-Akt), in steady-state S100A6KO HSCs (Fig. 4g, h). We confirmed that S100A6 affects p-Akt but not the total Akt at the protein level (Fig S4d). Our western blot analysis showed a robust reduction of p-Akt levels in the KO, but this reduction was rescued in the SC79-treated KO (Fig. S4d, e). Although PI3K is the major mode of Akt activation, our data demonstrated that both PI3K and PTEN activities remained unchanged (Fig. S4f, g). Moreover, our RNA-seq data did not detect any difference in *Pi3kr1* and *Pten* transcripts (data not shown). Progenitor colony formation in methylcellulose (CFU) assays displayed a reduction in colony-forming activity in S100A6KO BM cells. Interestingly, Akt activator SC79 rescued the colony formation activity defect in the KO BM cells (Fig. 4i), suggesting that p-Akt is a target of S100A6. Our findings suggest that S100A6-deficient HSC can be rescued by restoring the Akt pathway in murine HSCs, in which our data provide the underlying mechanism on how S100A6 regulates self-renewal of the HSCs through the Akt activation pathway (also in Fig. 3c, below).

S100A6 is known for its intra- and extracellular functions and the S100A6 protein is secreted upon a Ca^2+^ signal. S100A6 can form polymers and bind to the receptor for advanced glycation end-products [28]. To determine whether the effect of S100A6 on LT-HSCs is driven by niche modulations [29], we performed reverse transplantation experiments, where WT cells were transplanted into S100A6WT and S100A6KO mice. There was no difference in blood reconstitution and lineage output 16 weeks after reverse transplantation between groups of WT and KO transplanted HSCs into S100A6-deficient mice (Fig. S4h, i). To confirm this, we also tried to neutralize the S100A6-Akt signaling in WT HSCs by using blocking antibody against calcyclin antibody (H-55). H-55 inhibited the colony formation of the WT HSCs, but not of WT HSCs in the absence of H-55 (Fig. S4j). We therefore ruled out the possibility of S100A6 being a niche factor. Our findings demonstrate that S100A6 is a critical regulator of LT-HSCs repopulation capacity.

### S100A6 positively regulates intracellular calcium (Ca_i_^2+^) and S100A6-calcium uptake is p-Akt dependent

**Fig. 5:**
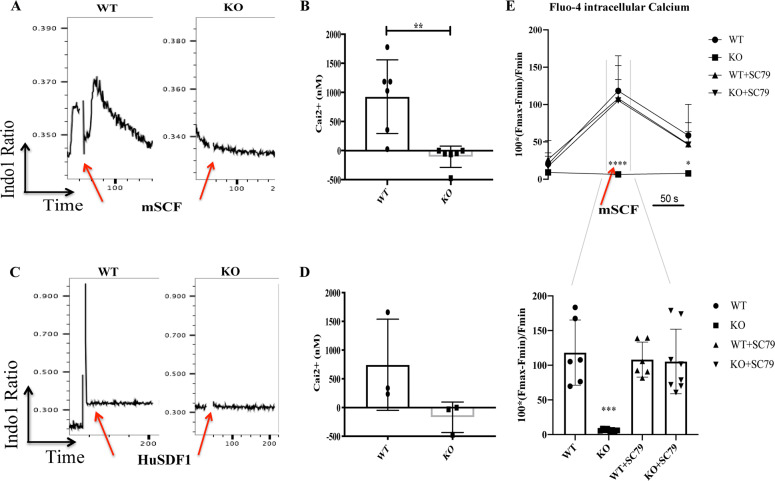
S100A6 null HSCs failed to mobilize intracellular calcium flux upon stimulation and S100A6-calcium flux is p-Akt dependent. Time gating before (first 100,000 cells were recorded) and immediately after addition of mSCF (**a**) or HuSDF-1 (**c**). The c-kit-enriched cells were exposed to mSCF (**a**) or HuSDF-1 (**c**) and the data show that only the HSC in the WT responded to both stimuli but not the S100A6 null cells as indicated by a change of the ratio between the violet (Ca^2+^ bound) and blue (free) fluorescence of Indo1. **b**, **d** Histograms show the changes in [Ca^2+^]_i,_ cytoplasmic Ca^2+^ evoked by mSCF and HuSDF-1, respectively. Error bars represent SD in S100A6WT and KO c-kit-enriched bone marrow cells stained with Indo1 gated on CD150^+^CD48^−^CD34^−^Flt^−^ (**b** *n* = 6; **d** *n* = 3; *p < 0.05; Mann–Whitney *U* test). Experiments were repeated three times. **e** (above) The corresponding change in [Ca^2+^]_i_ measured by Fluo-4 AM fluorescence intensity before, during and after the stimulation with mSCF over 50 s gated on CD150^+^CD48^−^CD34^−^Flt^−^. (below) Trace the period of mSCF application and total samples that respond upon mSCF stimulation with or without SC79 (WT, WT + SC79 *n* = 6; KO, KO + SC79 *n* = 8); *** or ****p < 0.0001; analyzed by multiple *t*-test (above); Mann–Whitney *U* test (below), which are representative of three independent experiments.

We asked whether p-Akt is the main downstream target of S100A6 upon upstream stimuli that activate S100A6-Ca_i_^2+^ signaling. To confirm our transcriptome finding and given that S100A6 is a calcium-binding protein, we interrogated the involvement of extrinsic factors that act on S100A6-Ca_i_^2+^ signaling. We know that stromal cell-derived factor (SDF-1) and SCF induce strong Ca_i_^2+^ flux [30–32]. Therefore, we examined the participation of Ca_i_^2+^ in S100A6 HSCs by stimulating WT and KO c-kit-enriched cells with the cytokine murine stem cell factor (mSCF) or the chemokine human stromal cell-derived factor 1 (HuSDF-1). Indeed, mSCF-induced Ca_i_^2+^ was decreased significantly in S100A6KO compared with S100A6WT in the LT-HSC population (Fig. 5a, b). Furthermore, S100A6KO also reduced HuSDF-1-induced Ca_i_^2+^ in the LT-HSC population (Fig. 5c, d). Therefore, the effects of SCF or SDF-1 signaling pathways on Ca_i_^2+^ require S100A6 expression in LT-HSC regulation. Our data demonstrated that S100A6 acts on Akt pathway (Fig. 4g–i) and it is known that both SCF and SDF-1 are Akt activators [9, 10]. Our data strongly indicate that in the absence of S100A6, SCF/SDF-1 fail to stimulate calcium flux, and impaired of Ca_i_^2+^ flux leads to a downregulation of the Akt pathway.

Similarly, we applied another calcium indicator, Fluo-4 to measure the median fluorescence intensity (MFI) of Ca_i_^2+^ upon binding Ca^2+^. When mSCF was added to the cells, MFI increased from a resting value (Fmin) to the peak. The peak (Fmax) occurred after onset of the mSCF application, then Ca_i_^2+^ decreased to Fmin, where the calcium flux lasts for 50 s. We observed increased calcium binding in the WT, but not in the KO (Fig. 5e, above). When we treated the c-kit-enriched cells with the SC79 on both the WT and KO cells, the calcium binding in the KO showed similar MFI as the WT (Fig. 5e, below), suggesting that S100A6-Ca_i_^2+^ uptake is p-Akt dependent. Together, we reason that S100A6-Ca_i_^2+^ acts specifically to stimulate the p-Akt pathway in murine HSC.

### S100A6KO HSCs do not respond to 5-fluorouracil (5FU) chemotoxic stress and S100A6 governs the Hsp90 chaperoning system through the p-Akt pathway

Our unpublished RNA-seq data indicated that S100A6 expression increased specifically after 5FU stress in the WT LT-HSC, but not in the S100A6KO. S100A6 promotes cancer cell proliferation [33]. In order to understand whether the deficiency of S100A6KO in self-renewal and reconstitution may be due to increased cycling and may lead to HSC exhaustion, we performed qRT-PCR on LT-HSC (LSKCD150^+^CD48^−^CD34^−^Flt3^−^) cells and we did not see any difference in most of the cell cycle genes, except a significant reduction in the *Ccna2* transcript (Fig. S5a). We assessed cell cycle status of HSCs using Ki67/DAPI staining on c-kit-enriched bone marrow cells. We found no difference in cycling status of S100A6KO HSCs (Fig. S5b, c). Furthermore, our RNA-seq data showed no significant differences in cell cycles gene expression between WT and KO LT-HSCs (data not shown).

**Fig. 6:**
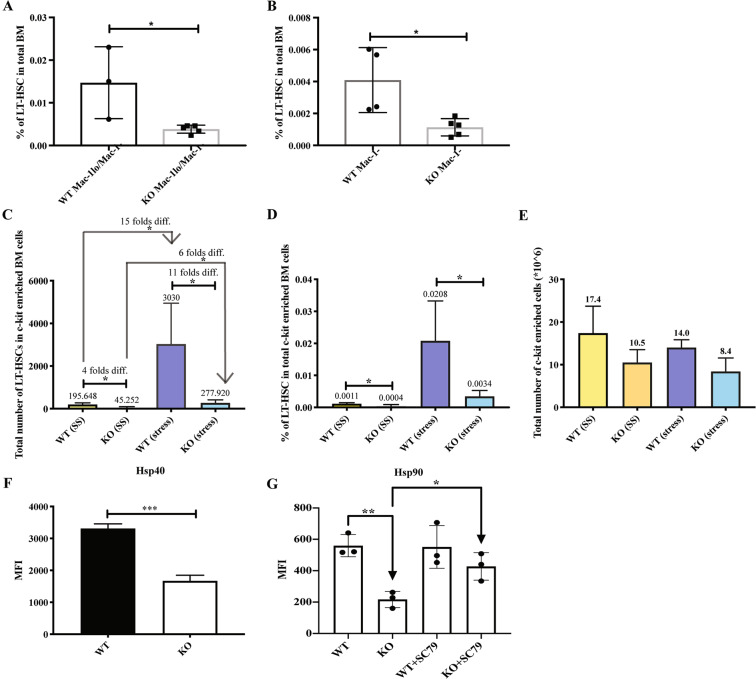
The LT-HSCs in S100A6KO mice were unable to respond to 5FU chemotoxic stress and the chaperonin Hsp90 activity of S100A6KO was restored by Akt activator. **a** Frequencies of LT-HSC (LSKMac^lo^/Mac^−^CD150^+^CD48^−^) in total bone marrow 12 days after 5FU administration (WT *n* = 3, KO *n* = 5). **b** Frequencies of LT-HSC (LSKMac^−^CD150^+^CD48^−^) in total bone marrow 12 days after 5FU administration (WT *n* = 4, KO *n* = 5). **c**, **d** Frequencies and total number of cells from a stringent gating of LT-HSCs (LSKMac^−^CD150^+^CD48^−^ESAM^+^CD9^Hi^) in c-kit-enriched bone marrow cells 12 days after 5FU administration. Steady state (SS), stress introduced with 5FU administration (S) (SS (WT, KO) *n* = 4; S (WT, KO) *n* = 3). **e** Absolute numbers of total c-kit-enriched bone marrow cells 12 days after 5FU administration (SS (WT, KO) *n* = 4; S (WT, KO) *n* = 3) (*p < 0.05; **p < 0.001; *** or ****p < 0.0001; all data dissected by unpaired two-sided *t*-test and Mann–Whitney *U* test). **f** Intracellular staining of Hsp40 in LT-HSCs (*n* = 3). **g** Intracellular staining of Hsp90 in LT-HSCs. Akt activator, SC79 restored Hsp90 activity in the KO (**f**, **g**) (*n* = 3). Data represent mean values from independent experiments ± SD. *p < 0.05; **p < 0.001, gated on CD150^+^CD48^−^CD34^−^Flt^−^, analyzed by unpaired two-sided *t*-test. The variance found inside values in a single group was smaller than the variance caused by interactions between different samples (one-way ANOVA, *p* = 0.006).

To functionally test the cell cycle status of S100A6KO, we created transient hematopoietic stress by intravenously injecting the myeloablative agent 5FU into WT and KO mice. HSCs are stimulated to cycle rapidly after treatment with 5FU [34]. For analysis of 5FU treated BM, Mac-1 (CD11b) was not included in the lineage cocktail antibody for gating out lineage negative cells, as expression of the integrin Mac-1 is known to increase after 5FU treatment [35]. The S100A6WT and KO mice survived the single injection of 5FU regimen (data not shown), however, the KO mice displayed an extremely low frequency of LT-HSCs (LSKMac-1^lo^/Mac-1^−^CD150^+^CD48^−^) (Fig. 6a) and LSKMac-1^−^CD150^+^CD48^−^ cells (Fig. 6b) in total bone marrow 12 days after 5FU administration. Given these surface markers (LSKMac-1^−^CD150^+^CD48^−^) were sufficient to interpret our observation, we further applied the more strict gating strategy as shown (Fig. 2h). Interestingly, while the myelosuppression induced by the 5FU after 12 days led to an increase in LT-HSCs (LSKMac-1^−^CD150^+^CD48^−^ESAM^+^CD9^Hi^) in WT cells, the absolute total cell numbers and frequency of KO LT-HSC cells were drastically reduced (Fig. 6c, d). Total cellularity decreased in S100A6KO mice with or without 5FU stress (Fig. 6e). These data show that in the absence of S100A6, the LT-HSCs exhibit an impaired response to the chemotoxic stress.

Our next question was to understand the role of S100A6 under physiological stress condition in LT-HSC. It is known that S100A6 expression is increased after thermal stress [13] and we therefore investigated the chaperonin protein activity in the absence of S100A6. Hsp90 is an essential molecular chaperone that assists in protein folding quality control [36, 37]. In normal cells, Hsp90 is found in mitochondria, and is robustly present in the cytosol [38]. Hsp90 binds to Akt in vivo, but disruption of this complex formation increases apoptotic cells [39, 40]. S100A6 is also bound weakly to GST-truncated Hsp90 in vitro [41]. S100A6 associates with Hsp70/Hsp90-organizing protein (Hop) through the tetratricopeptide domains and regulates the Hop complex formation. Endogenous S100A6 modulates Hsp70-Hop-Hsp90 heterocomplex assembly in vivo in a Ca^2+^-regulated manner [18]. Although we found no difference in the MFI of the chaperonin protein Hsp70 in S100A6KO HSCs (Fig. S6a), we observed a significant reduction in the MFI of Hsp40 (Fig. 6f) and Hsp90 (Fig. 6g) in these HSCs, reaffirming that S100A6 regulates Hsp40-Hop-Hsp90 heterocomplex in the hematopoietic system. We next asked if Hsp90 activity could be rescued by reactivating the Akt pathway in an S100A6-dependent manner. Our results show that SC79 restored Hsp90 activity in the S100A6KO HSCs (Fig. 6g). Taken together, we therefore suggest that S100A6 affects the Hsp90 chaperoning system through connection with the Akt activation pathway. This result confirms that decreased intracellular Hsp90 protein is due to the deficiency of S100A6 (Table 1, above).

### S100A6/Akt activation pathway is required for efficient mitochondrial function in HSCs

**Fig. 7:**
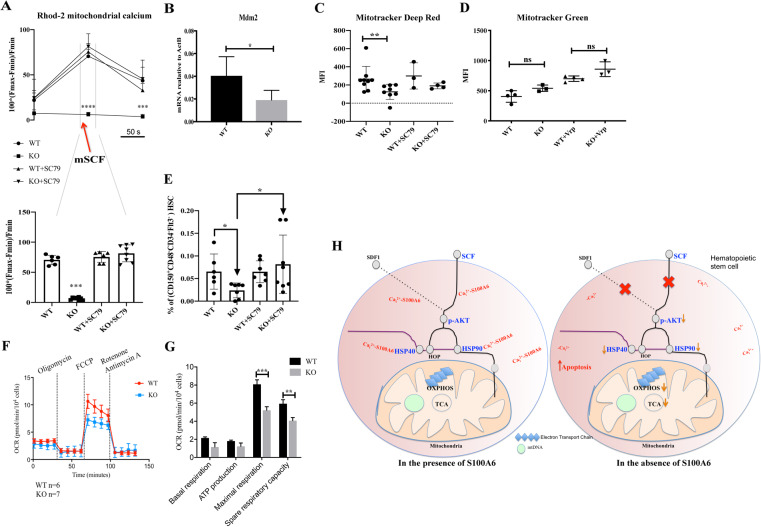
S100A6 regulates mitochondrial calcium levels and mitochondrial respiratory capacity; Akt activator SC79 molecule restores S100A6KO LT-HSC compartment. **a** (above) The corresponding change measured by Rhod-2 AM fluorescence intensity in mitochondrial calcium before, during, and after the stimulation with mSCF over 50 s, gated on CD150^+^CD48^−^CD34^−^Flt^−^. (below) Trace the period of mSCF application on Rhod-2 fluorescence and total samples that respond upon mSCF stimulation phase (WT, WT + SC79 *n* = 6; KO, KO + SC79 *n* = 8); *** or **** *p* < 0.0001; analyzed by multiple *t*-test (above); Mann–Whitney *U* test (below). **b** qRT-PCR analysis for *Mdm2* mRNA expression and relative mRNA expression is normalized to *ActB* expression levels, and mean ± SD is shown (*n* = 6). Intracellular staining of Mitotracker deep red (**c**) and Mitotracker green (**d**) in S100A6WT and KO HSCs for 30 min and analyzed by flow cytometry gated on CD150^+^CD48^−^CD34^−^Flt^−^. **c** SC79 added on c-kit-enriched cells for 20 min at room temperature, before Mitotracker deep red staining. **e** Frequency of S100A6KO LT-HSC (LSK CD150^+^CD48^−^CD34^−^Flt3^−^) from total c-kit-enriched BM cells is restored after Akt activator SC79 treatment (*p < 0.05; analyzed by Mann–Whitney *U* test). **f** Oxygen consumption rate (OCR) trace was determined using a Seahorse XF96 Analyzer. **g** Maximum respiratory capacity and reserve respiratory capacity decreased in S100A6 null cells. *p < 0.05; * *p < 0.001; ***p < 0.000 as determined by multiple *t*-test. Bars represent mean ± SD (*n* = 7). **h** Summary of S100A6 regulation of mitochondria oxidative phosphorylation through Akt and Hsp90 interaction in mouse HSC. All data represent mean values from independent experiments ± SD. *p < 0.05, analyzed by unpaired *t*-test.

Next, we asked whether impaired S100A6-Ca_i_^2+^ sensing, Akt signaling, and Hsp90 chaperoning system lead to LT-HSC metabolic dysfunction. To investigate whether mitochondrial Ca^2+^ homeostasis during Ca^2+^ influx and Ca^2+^ release in HSC is S100A6 and p-Akt dependent, we applied Ca^2+^ sensitive fluorescent indicator Rhod-2 to monitor mitochondrial Ca^2+^ (Ca_m_^2+^) concentration. Then we stimulated c-kit-enriched cells with mSCF, which raises Ca_m_^2+^ from Fmin to the peak, then returning to its Fmin, for a total of 50 s. Similarly, as we have noticed in Ca_i_^2+^ (Fig. 5e), the S100A6KO showed no uptake of Ca_m_^2+^ (Fig. 7a, above). Amazingly, SC79 reactivated the S100A6KO HSC and allowed uptake of Ca_m_^2+^ to resemble the WT (Fig. 7a, below).

S100A6 binds to mouse double minute 2 homolog (Mdm2) [42] and Mdm2 is a mitochondrial marker [43], we reasoned that in the absence of S100A6-Ca_i_^2+^ axis might negatively regulate the Akt downstream target Mdm2 pathway in the maintenance of HSC. Our qRT-PCR confirmed that the expression of *Mdm2* was reduced in S100A6KO HSCs (Fig. 7b). Mdm2 was noticed as one of the significant downregulated genes in the volcano plot (Fig. 3a). However, there was no difference in the *Cdkn1a (p21)* transcript (a downstream target gene of p53, Fig. S5a), between the S100A6WT and KO HSCs.

To investigate the effect of the S100A6KO on mitochondrial function, we used FACS to assess the levels of actively respiring mitochondria and total mitochondrial mass using mitochondria-specific labels (Mitotracker deep red and Mitotracker green, respectively). To exclude artifacts caused by active extrusion of the Mitotracker dyes, we carried out our experiment in the presence of verapamil. However, verapamil is also an inhibitor of calcium entry [6, 32]. We excluded verapamil in Fig. 7c to evaluate the physiological calcium effects on mitochondria activity. We observed that there was no obvious difference in the ratio before and after adding verapamil, between WT and KO (Fig. 7d). Our data showed that the S100A6KO exhibits a decrease in mitochondrial activity (Fig. 7c) but had similar mitochondria mass (Fig. 7d). Interestingly, SC79 revert the mitochondria activity in the S100A6KO (Fig. 7c). From the same cell samples, upon the received treatment on SC79, LT-HSC fraction from S100A6KO was recovered (Fig. 7e), and this suggests that we are able to rescue the lost cells that we have observed in the KO (Fig. 2g).

Given that the activities of three key energetic mitochondrial dehydrogenases are stimulated by Ca^2+^ ions [44], we evaluated their levels of oxidative phosphorylation (OXPHOS) in HSC by measuring their oxygen consumption rate (OCR) with a Seahorse XF96 Analyzer. An equal number of LSK^+^ FACS sorted cells from WT and KO mice were evaluated. OCR was monitored at basal levels and after metabolic stress by addition of oligomycin, carbonilcyanide p-triflouromethoxyphenylhydrazone (FCCP), and antimycin A/rotenone in succession. S100A6KO LSK^+^ cells showed slightly lower levels of basal OCR and ATP production, as compared with their WT counterparts (Fig. 7f, g). Significantly, in the absence of S100A6, LSK^+^ cells showed robustly reduced levels of maximal respiration and spare respiratory capacity, demonstrating impairment in the ability of these cells to perform OXPHOS (Fig. 7f). Low transcripts levels of citrate synthase (Fig. 3a) and low protein level of IDH2 (Fig. 3d) suggest that the TCA cycle is affected in S100A6KO LT-HSCs and therefore contributes further to impaired function of mitochondria to produce cellular energy through OXPHOS in the electron transport chain (Fig. 7f, g). Our data strongly suggest that S100A6KO negatively affects mitochondrial respiration in LT-HSCs, and an intact S100A6-Ca_i_^2+^ influx through Akt activation pathway is essential for mitochondrial energy production.

## Discussion

In this study, we characterized an important chaperone-like calcium sensor protein, S100A6, and its role in regulating HSCs. We have demonstrated that S100A6 plays an essential role in the maintenance of steady-state HSCs. By performing genetic disruption of the S100A6 gene in mouse, our bulk transcriptomic and proteomic data demonstrated that S100A6KO HSCs have decreased levels of Ca_i_^2+^, Akt phosphorylates targets, mitochondrial mRNA, and reduction of Hsp90 protein levels. Our serial transplantation assays revealed that S100A6 is critical for HSC self-renewal, and calcium uptake for both intracellular and mitochondrial functions, indicating a key role for an intact calcium-dependent S100A6 signaling for regulation of HSC fate options. Our data explain how S100A6 is a direct responder of Ca^2+^ signaling on intracellular and mitochondrial HSC, and transmits a signal by cytokine SCF or chemokine SDF-1 to regulate stem cell activities. Our findings also show how Ca_i_^2+^, Ca_m_^2+^, and HSC are interconnected. S100A6 maintains colony-forming capacity and the LT-HSC population through regulation of the Akt activation pathway in the hematopoietic system. Interestingly, we have uncovered an important role of S100A6 as a prerequisite regulator of intact mitochondrial metabolism by maintaining OXPHOS activity in the steady state. Our 5FU data indicated that S100A6KO HSCs could not respond to chemotoxic stress, indicating that the role of S100A6 is to protect cells from stress through collaboration with the Akt-Hsp90 cheperoning system for proper protein quality.

Our results showed that S100A6KO reduces myeloid output under steady-state conditions. Plscr1 is required for normal myelopoiesis and reported to collaborate with Ca^2+^ to develop acute myeloid leukemia [45, 46]. We reasoned that in the absence of S100A6, disrupted Plscr1 contributes to decreased myeloid output. Our result showed that S100A6 maintains the myeloid lineage, and in agreement with a previous study, S100A6 is expressed in myeloid cells [47].

A previous study has shown that S100A6 translocation was found to be distinct and dependent on actin-stress fibers, but not dependent on the classic Golgi-ER pathway in response to an increase in Ca_i_^2+^ levels [48]. Our intracellular staining analysis of Grp94 (Fig. S6b) and Grp75 (Fig. S6c) demonstrated that there was no significant difference between WT and KO. Grp94 and Grp75 are ER proteins [49, 50]. Moreover, our data showed no difference of transcript levels of S100A8 and S100A9 in the absence of S100A6 (Fig. 1g, h). S100A8 and S100A9 are known to be normally associated to Golgi-ER [47]. These findings suggest that S100A6 utilizes distinct translocation pathways, which leads the protein to certain subcellular compartments in order to perform their physiological tasks. This suggests that S100A6 is not acting on the classical Golgi-ER pathway.

In Fig. 7h, a map illustrates the complexity of the S100A6 in regulating HSC. It depicts the role of S100A6 in murine HSCs and how S100A6-Ca_i_^2+^-dependent regulation affects mitochondrial metabolism through various intermediates. In the presence of S100A6-Ca_i_^2+^, stimulation with SCF or SDF-1 increases Ca_i_^2+^ concentration, thereby recruits S100A6 to interact with p-Akt and activates the p-Akt-Hsp90 pathway. S100A6KO do not alter the expression of PI3K, the upstream activator of p-Akt in mouse HSC (Fig. S4f, g), likewise the *Pi3kr1* and *Pten* transcripts were not changed either (Fig. S5a). Akt contains a pleckstrin homology domain (PHD) [51] and our RNA-seq data show that the *Plekhf2* transcript is downregulated in S100A6KO HSC (data not shown). We picture that S100A6 may interact with Akt through PHD hence facilitating Akt translocation to the membrane upon SCF/c-kit stimulation. S100A6-Ca_i_^2+^ activated p-Akt pathway also regulates chaperoning protein members Hsp90 and Hsp40 for proper protein folding (Fig. 6f, g). S100A6 protein maintains mitochondrial calcium and respiration functions in the WT. In contrast, stimulation of Ca_i_^2+^ transient activities by SCF or SDF-1 fails to be detected in the absence of S100A6, thus the p-Akt pathway is shut off. In addition, the Hsp90 chaperoning system is downregulated. Given our findings that S100A6-deficient cells have decreased signatures of the molecules in the Hsp90 chaperoning system, which leads to detrimental to HSC integrity [52], OXPHOS/metabolism dysfunction is provoked by the loss of S100A6. As a consequence of all the above, HSCs in the S100A6 null mice exhibit increased cellular apoptosis.

S100A6-mediated Akt regulation represents a target window for the development of new therapeutics specifically toward MLL-AF4 leukemia patients that may be treated with drugs that inhibit S100A6-expressing cancer cells.

## Supplementary information

Supplementary Material and Methods
