# Supplementary material for: S100A6 is a critical regulator of hematopoietic stem cells
Source: Leukemia. 2020 Jun 19;34(12):3323–37. doi: 10.1038/s41375-020-0901-2 (PMC7685984; doi:10.1038/s41375-020-0901-2)
Supplement: Supplementary file 1 — Supplementary Material and Methods [file 41375_2020_901_MOESM1_ESM.docx]

**Supplementary text**

**Suppl Fig 1. S100A6 is visualized in the LT-HSCs population and S100A6 trancripts are absent in the S100A6 null-vav-Cre**

**
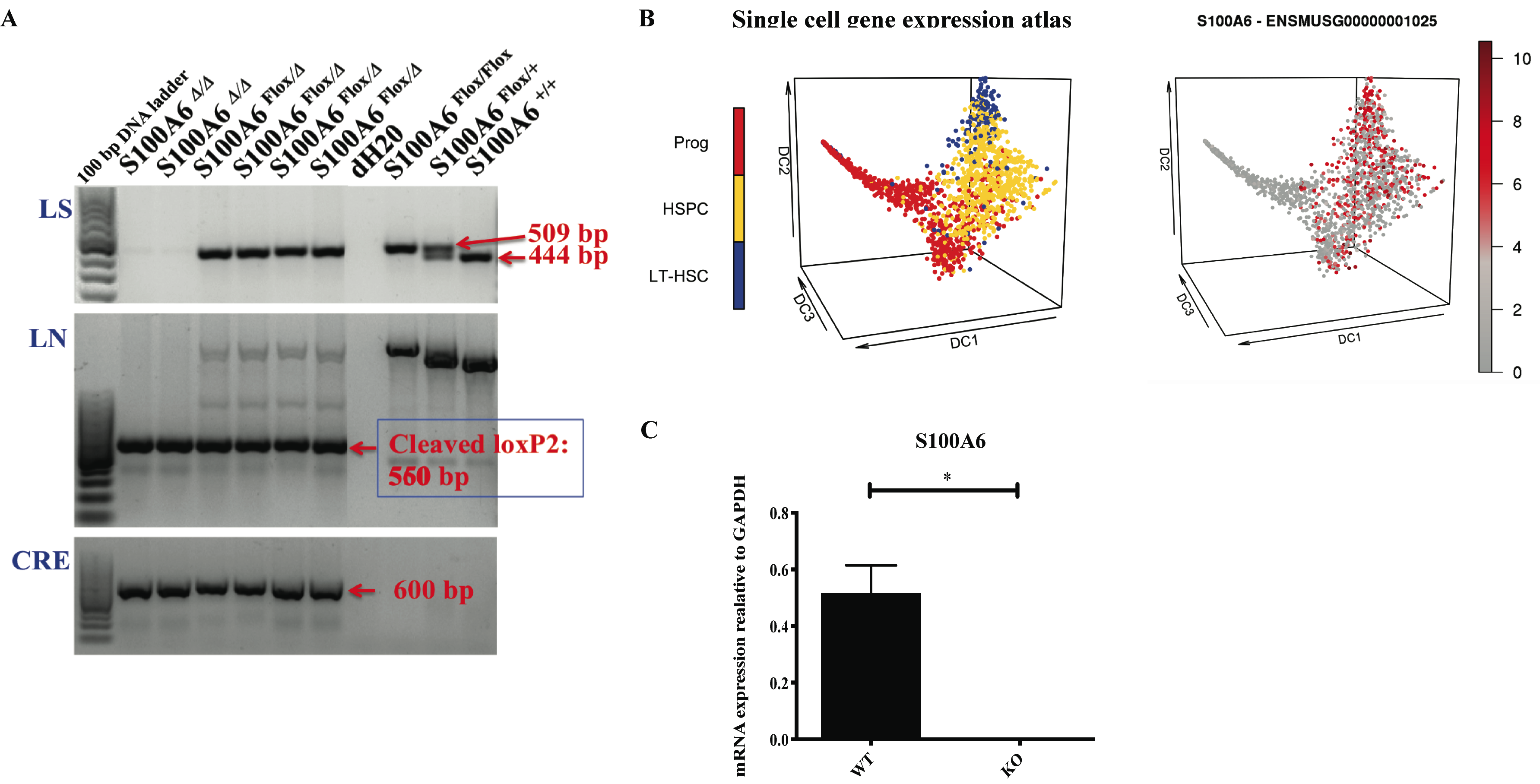
**

(A; top, middle, bottom) Genotyping analysis for *S100a6* gene inactivation in the mouse by the Cre/loxP-technique. (A, top) Amplification of genomic DNA for detection of loxP flanked exon 1-2 of the S100A6 gene, (primer sets: Lunds and Sdl2), and gives rise to either a double band for heterozygotes (of 444 bp and 509 bp) or a signal at 509 bp for animals homozygous for the loxP site. A double band depicts heterozygous genotype, one band at 509 bp only indicates homozygous loxP insertion. (A, middle) Cleaved exon 2-3 (loxP2), (primer sets: Lunds and Ndel2), to specifically detect deletion of the loxP-flanked exon 2-3 (loxP2) cleaved by Cre recombinase, indicating the real knockout. (A, bottom) Cre-transgene detection by PCR with genomic DNA as template. *S100a6^flox/flox^ or S100a6^flox/+^*(Wildtype*), VavCre;S100a6^ΔΔ^*(knockout). Primer pairs Lund3-Sdl2 (LS) are used to identify flox alleles; primer pairs Lunds-Ndel2 (LN) are intended to recognize the real excision of exon 2-3. See Figure 1b for location of primers.

(B) A web interface shows *S100A6* gene expression in LT-HSCs at single cell resolution. Cells from adult mouse bone marrow are captured in three gates: Prog (Lin^-^ Sca1^-^ c-Kit^+^), HSPC (Lin^-^ Sca1^+^ c-Kit^+^), LT-HSC (Lin^–^ c-Kit^+^ Sca1^+^ CD34^–^ Flk2^–^).

(C) qRT-PCR analysis for *S100a6* expression in c-kit enriched bone marrow. Each value is normalized to GAPDH expression and mean ± SD are shown (n=3, *p <0.05; analysed by unpaired two sided t-test).

**Suppl Fig 2. Schematic gating of LSK, CD150, CD48, CD34, Flt3 HSPC compartment, in steady state whole bone marrow cells**


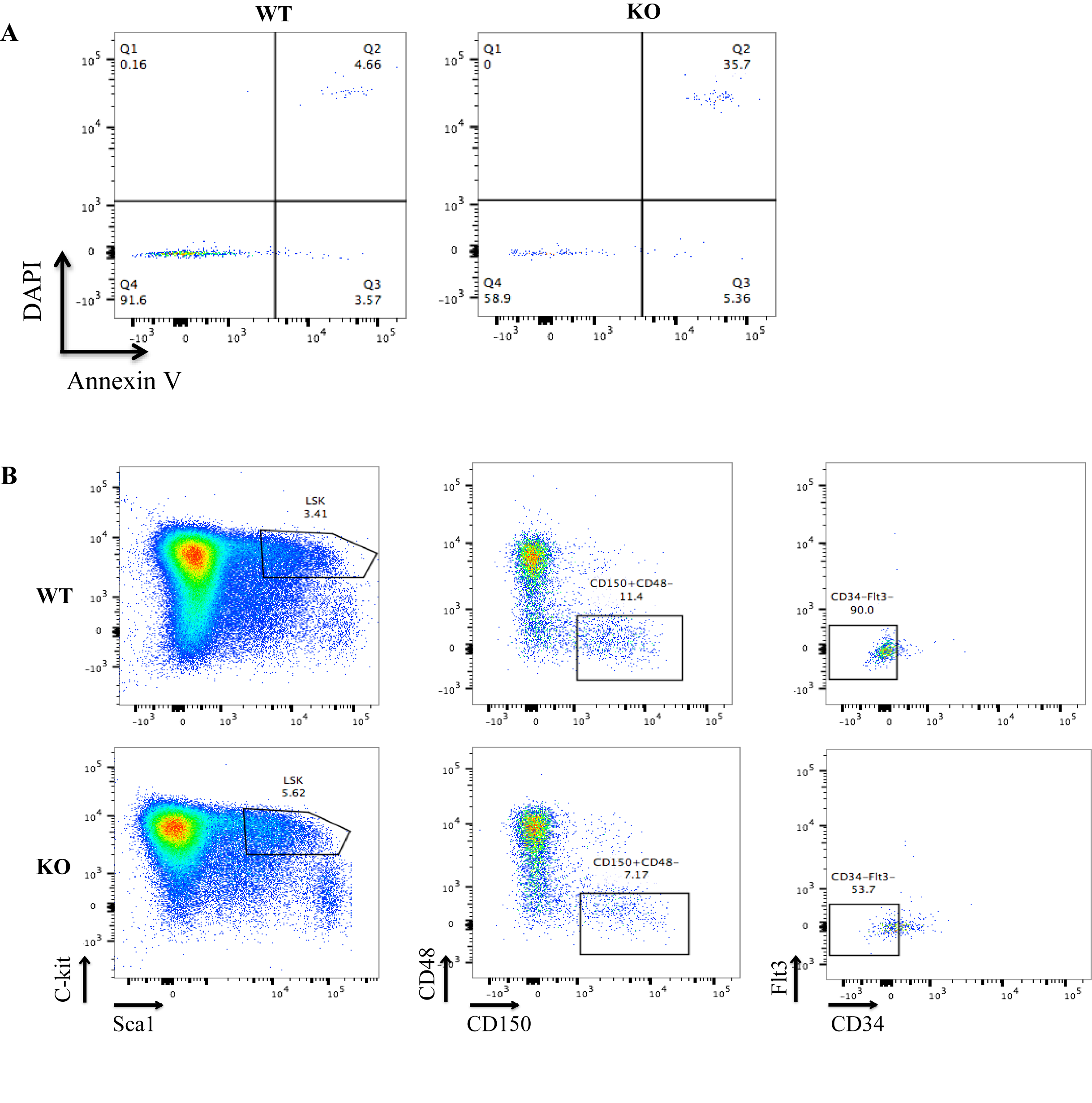


(A) FACS plots of the LSK CD150^+^ CD48^-^ HSPC compartment with apoptotic status assessed using Annexin V and DAPI.

Data represent mean values from independent experiments ± SD. * p<0.05, analysed by unpaired two sided t-test.

(B) Representative FACS plots of LT-HSC (CD150^+^CD48^-^CD34^-^Flt3^-^). S100A6KO had a robust reduction in the LT-HSC compartments compared to WT.

**Suppl Fig 3. Mitochondria transcripts are reduced in S100A6 null HSC**


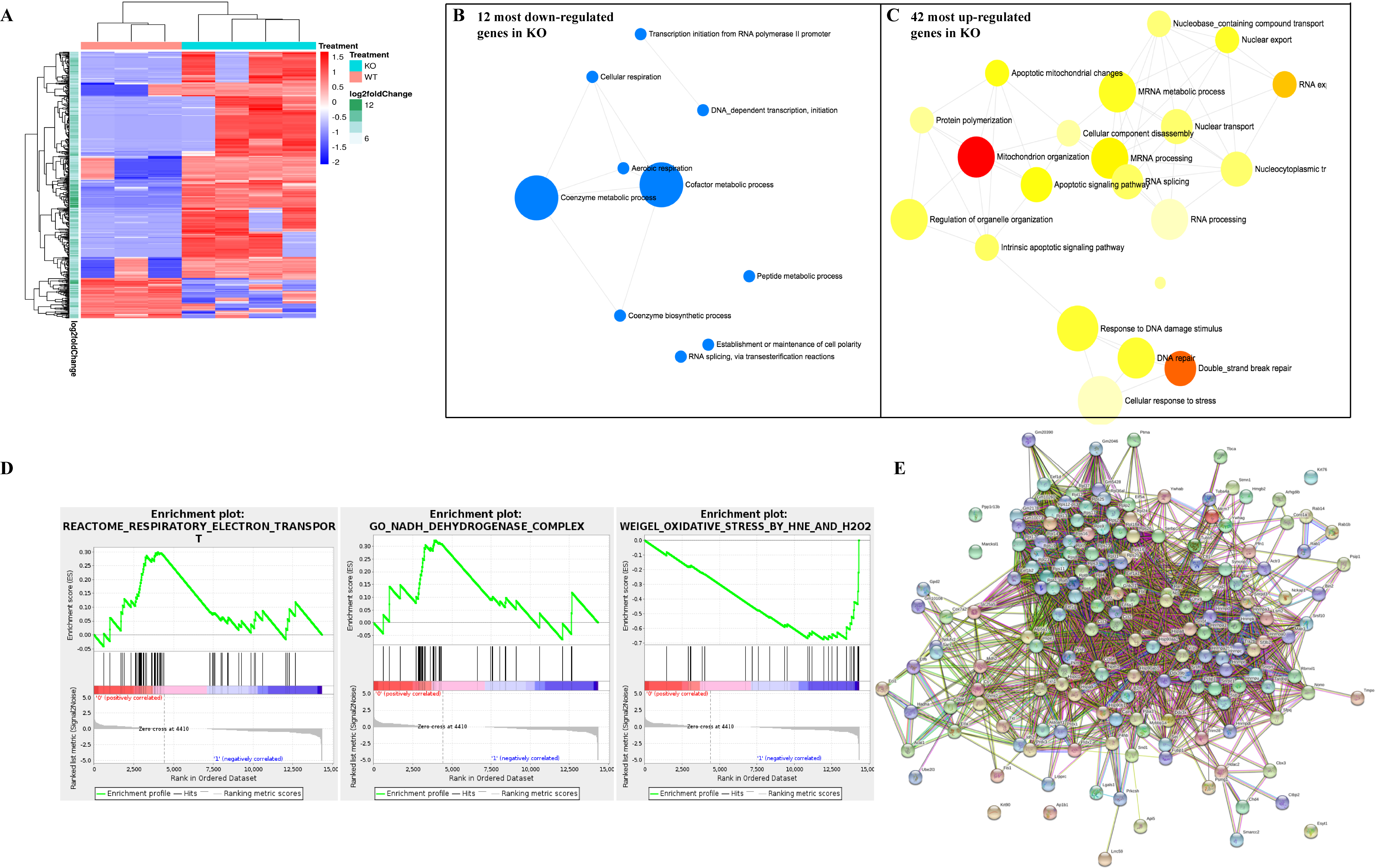


(A) Heatmap for 448 genes that have p<0.05.

(B, C) Gene Ontology enrichment analysis of biological process for a total of 54 genes (down-regulated (B) and up-regulated (C)) in S100A6 deficient samples derived from the heatmap (Fig. 3b), (accessed NetworkAnalyst 3.0).

(D, left) Gene set enrichment analysis (GSEA) results for NextSeq 500/550 v2 sequencing enriched in the WT compared to S100A6 null LT-HSCs signature. "Respiratory Electron Transport" represents genes upregulated in the WT but reduced in the S100A6KO. (NES: 1.3420614; nominal P value: 0.120229006; FDR q-value: 1.0; FWER P-value: 1.0);

(D, middle) "NADH Dehydrogenase Complex" represents genes upregulated in the WT but reduced in the S100A6KO. (NES: 1.4340184; nominal P value: 0.10359408; FDR q-value: 1.0; FWER P-value: 1.0).

(D, right) "Weigel oxidative stress by HNE and H_2_O_2_" represents genes downregulated in the WT but upregulated in the S100A6KO. (NES: -1.8449508; nominal P value: 0.0; FDR q-value: 1.0; FWER P-value: 0.512).

(E) The protein-protein networks view from STRING database showing the networks in the absence of S100A6

**Suppl Fig 4. S100A6 null mice have reduced p-Akt protein level, but exhibit normal hematopoiesis after transplantation of WT bone marrow cells and normal hematopoiesis after reverse transplantation**


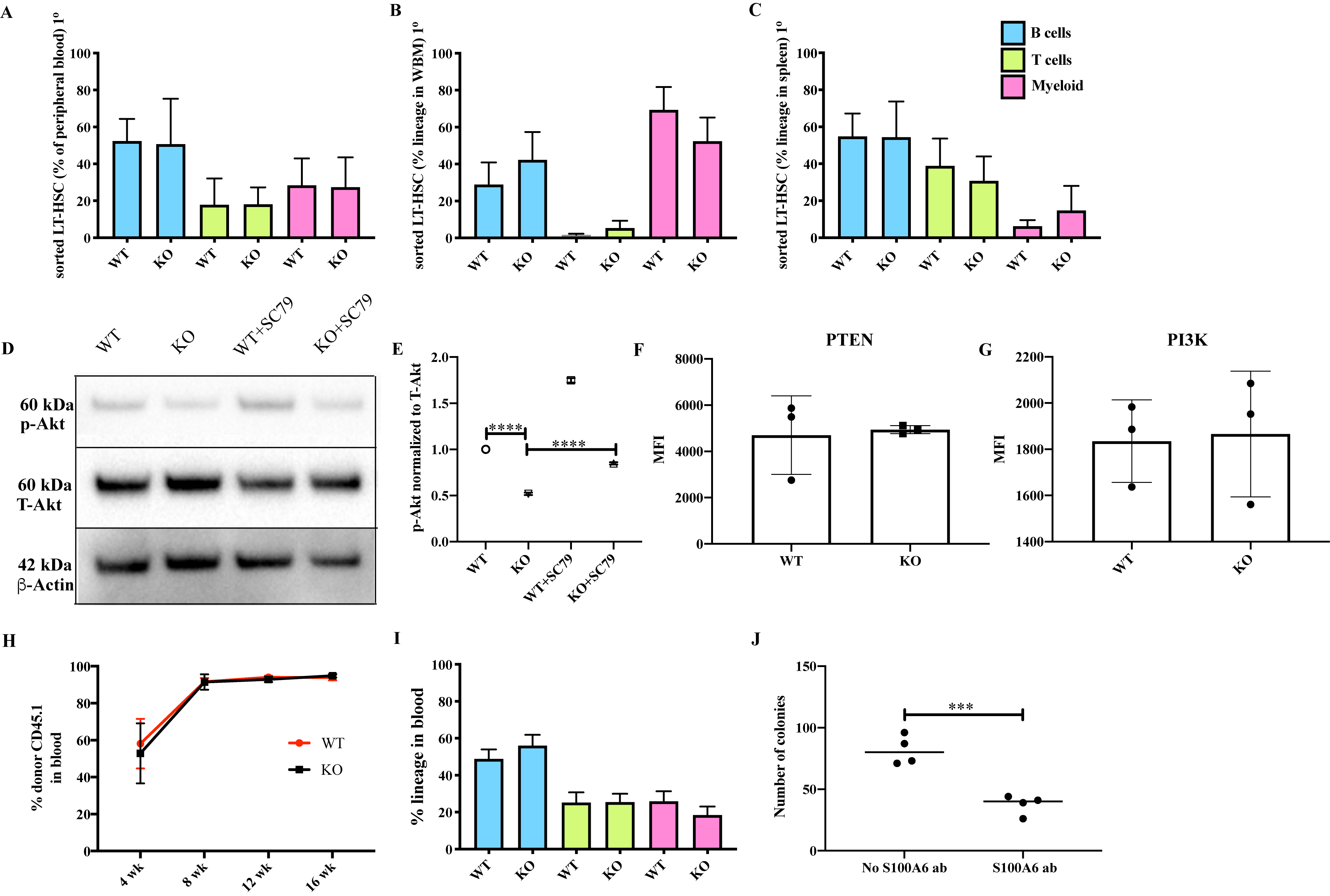


(A-C) LT-HSCs (CD150^+^CD48^-^CD34^-^Flt^-^) isolated from S100A6KO mice have normal hematopoiesis in blood (A), bone marrow (B) and spleen (C), as measured by flow cytometric analysis of surface markers CD3, B220, Mac1/Gr1.

(D) Representative western blot of three independent sorted LSK cells to check the expression levels of p-Akt, T-Akt, and β-actin. p-Akt was normalized to T-Akt. β-actin was used as a loading control.

(E) The relative expression of p-Akt and T-Akt was analyzed by ImageJ software. The dot graph represents the intensity ratio of p-Akt to T-Akt. WT was used as a reference band for samples of KO, WT+SC79 and KO+SC79 by ImageJ software.

(F) Intracellular staining of PTEN in LT-HSCs (CD150^+^CD48^-^CD34^-^Flt^-^);

(G) Intracellular staining of PI3K p110α in LT-HSCs.

(H) Immunophenotypic analysis of CD45.1/CD45.2 in blood showed similar cell engraftment in S100A6 WT and KO littermate recipients (16 weeks after reverse transplant).

(I) Normal hematopoiesis after 16 weeks reverse transplantation.

(J) CFU assay for purified WT HSCs cultured with or without calcyclin antibody (H-55). The number of colonies was significantly decreased only when WT was treated with H55, (n=4; *** p < 0.0001; analysed by a two-sided unpaired t-test).

All data represent mean values from independent experiments ± SD.

**Suppl Fig 5. S100A6KO have normal cell cycling**


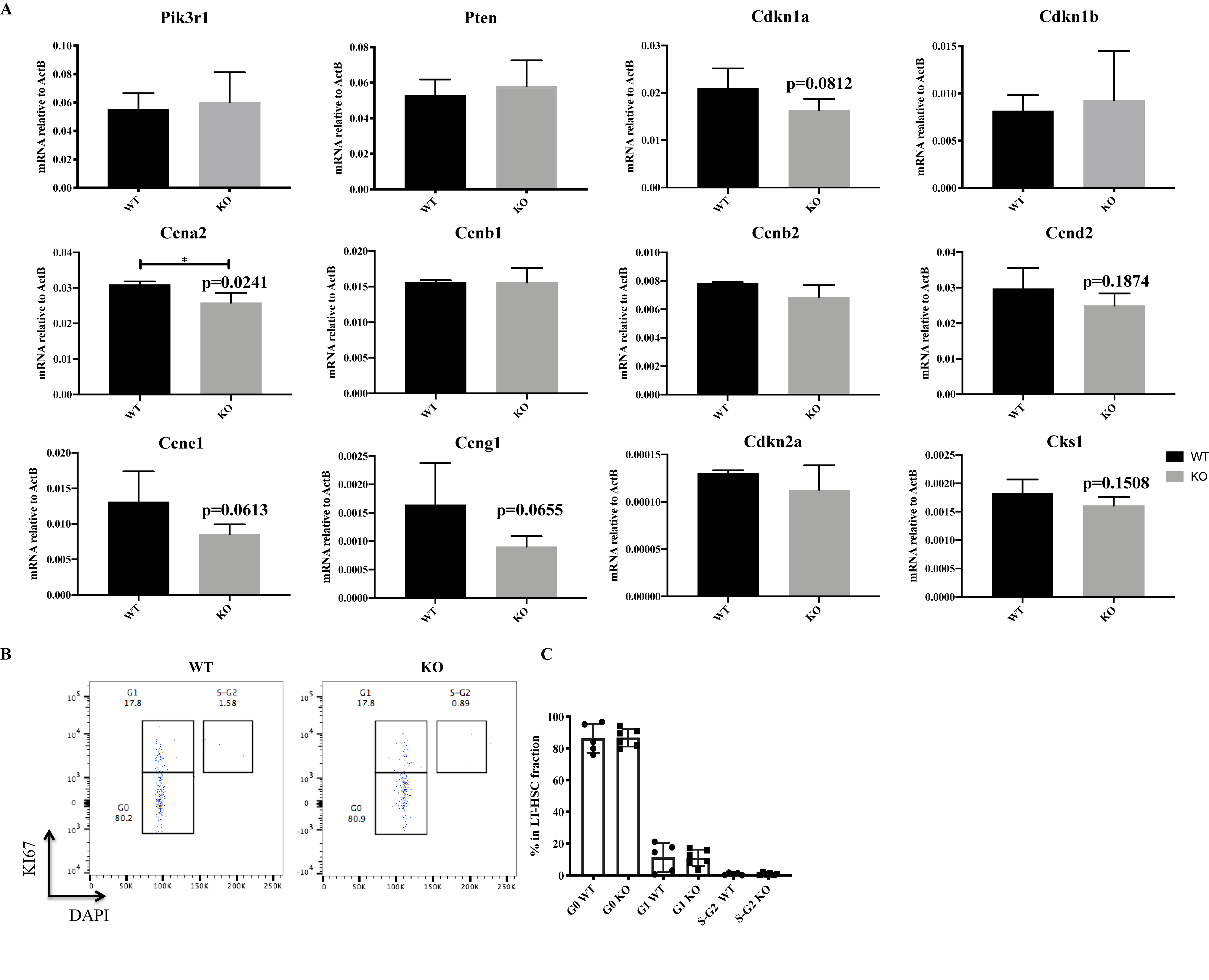


(A) mRNA levels of *Pik3r1* (n=9), *Pten* (n=9), *Cdkn1a* (n=3-5), *Cdkn1b* (n=9), *Ccna2* (n=5), *Ccnb1* (n=5), *Ccnb2* (n=5), *Ccnd2* (n=5), *Ccne1* (n=5), *Ccng1* (n=5), *Cdkn2a* (n=5), *Cks1* (n=5), assessed by qRT-PCR on LT-HSCs (CD150^+^CD48^-^CD34^-^Flt^-^); Results are the mean ± SD of triplicates. Each value is normalized to *ActB* expression. (*p <0.05; analysed by a two-sided unpaired t-test).

(B; left, right) FACS plots of LSK CD150^+^ CD48^-^ HSPC compartment with cell cycle status assessed using Ki67 and DAPI.

(C) Normal proliferation was observed in S100A6KO.

**Suppl Fig 6.** **S100A6 deficient HSC shows no significant difference in classical endoplasmic reticulum regulated proteins**


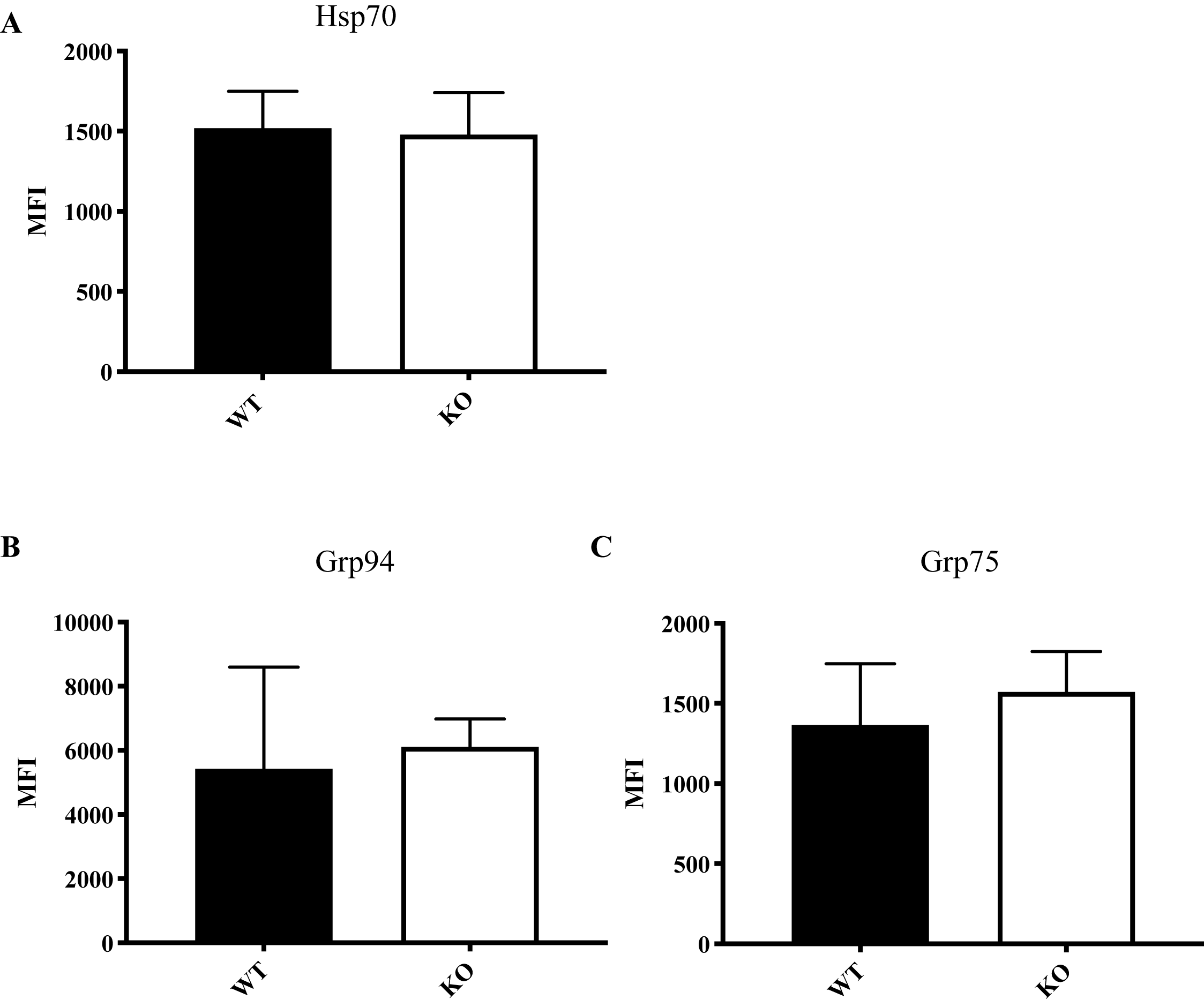


(A) Intracellular staining of Hsp70 in LT-HSCs (CD150^+^CD48^-^CD34^-^Flt^-^);

(B) Intracellular staining of Grp94 in LT-HSCs;

(C) Intracellular staining of Grp75 in LT-HSCs.

Results are the mean ± SD of triplicates, all data analysed by unpaired two sided t-test and Mann Whitney U-test.

**Primers for PCR screening**

NDEL1: 5’- GTC TGC CAG CTG ACC AGG -3’

NDEL2: 5’- CTG GAA CAC AGG CAG CTA CGG -3’

FLP1: 5’- CAC TGA TAT TGT AAG TAG TTT GC -3’

FLP2: 5’- CTA GTG CGA AGT AGT GAT CAG G -3'

LUND3: 5’- CAT TGG GAT CCA AGT TTT CGG AGC -3’

SDL2: 5’- ATG CCA TGA CTG ATC ACT GGG CTA -3’

FRTAN9: 5’- CGG CTT AAG TGT ACA CGC GTA CTA GT –3’

A2: 5’- GGA GAT GAG GCC ACA GTA ACT GTA G –3’

**Supplementary Materials and methods**

**Long-term competitive repopulation assays**

Serial transplantation assays were performed to determine the long-term engraftment capability of S100A6 *in vivo*. Bone marrow cells were harvested by crushing the femurs from 8-10 week old S100A6 KO and littermate control mice (Ly5.2). For 1^o^ transplantations, 200,000 cells from the WT and KO cells were competitively transplanted together with 200,000 bone marrow cells from the supporting mice (Ly5.1) into lethally irradiated (900 cGy) recipient mice (Ly5.1). After 16 weeks, 2^o^ transplantations were carried out by transplanting 2x10^6^ donor derived WT or KO bone marrow cells into newly lethally irradiated recipients. For the following 3^o^ transplantations, 20x10^6^ donor derived cells from WT or KO mice were transplanted into lethally irradiated recipients. Serial transplantations were repeated three times. Every 4 weeks after transplantation, peripheral blood was collected from recipient mice and analyzed for chimerism and lineage distribution. Percent chimerism was defined as (percent Ly5.1^+^ test donor cells) (100)/(percent Ly5.1^+^ test donor cells + percent Ly5.2^+^ competitor cells).

**Intracellular staining of heat shock proteins**

Approximately 2x10^6^ c-kit enriched cells were fixed with 100 μl BD Fix/Perm solution in an eppendorf tube, gently mixed by flicking the tube and incubated for 20 min at 4°C. After fixation, 1 ml of 1x wash/perm solution was added and cells were spun down at 500x g for 5 min. Wash/perm was discarded and 100 μl of 0.25% TritonX-100 (diluted in wash/perm solution) added to the pellet and incubated for 30 min at 4°C. Then, 1 ml wash/perm was added and spun at 500x g for 5 min.

Fixed cells were stained with the following primary antibodies for 30 min at 4°C; anti-GRP94 (EPR3988, Abcam, Cambridge, UK), -GRP75 (D13H4), -HSP70, -HSP40 (C64B4), -HSP90 (C45G5), PI3K p110α (C73F8), PTEN (138G6), phospho-Akt (Ser473, 193H12), phospho-4E-BP1 (Thr37/46, 236B4), rabbit IgG isotype control (DA1E) (all Cell Signaling Technology), Alexa Fluor® 488 conjugated rabbit anti-goat IgG (Abcam, Cambridge, UK) was used as a secondary antibody. Analyses were performed using FACS LSRII^TM^.

**RNA sequencing (RNA-seq)**

We performed messenger RNA (mRNA) sequencing in S100A6 WT and KO mice. WT and KO mice with matched littermates, blinded gender were used. RNA from 500 (LSKCD150^+^CD48^-^Flt3^-^CD34^-^) sorted cells from each mouse (WT and KO) were processed using the SMART-Seq® v4 Ultra® Low Input RNA Kit (Clontech, Göteborg, Sweden) to obtain cDNA. The obtained cDNA was purified using AMPURE XP beads (Agilent). cDNA was amplified with 20-22 PCR cycles and quantified on a Bioanalyzer High Sensitivity DNA chip (Agilent). Dual-indexed cDNA sequencing libraries were constructed using the Nextera XT DNA Library Preparation Kit (Illumina, San Diego, CA, USA) in order to fragment and tag the cDNA with sequencing adapters for the barcoding of each sample. The cDNA library was sequenced using NextSeq 500/550 v2 sequencing reagent kits with a read length of 76 bases in paired-end mode. The WT and KO conditions were assessed in sextuplicates. The sequenced reads were aligned to the mouse reference genome (GRCm38/mm10) using HISAT2 (v2.0.5) [1]. Duplicated reads were marked and filtered out using Picard MarkDuplicates tool (v8.25) (http://broadinstitute.github.io/picard/). Fragments aligned to the mouse genes (Ensembl GRCm38.91) were quantified using the featureCounts tool [2]. Differential gene expression analysis was performed using R (v3.2.3) package limma (v3.26.9) [3]. Briefly, raw read counts per gene were transformed to log scale and mean-variance trends were estimated using VOOM [4]. The mean-variance estimates were incorporated to linear model fitting to identify differentially expressed genes. Gene set enrichment analysis was performed using Gene Set Enrichment Analysis (GSEA) software (v3.0) [5].

**Protein extraction, proteolytic digestion and chemical labeling**

Sorted cells (LSKCD150^+^CD48^-^Flt3^-^CD34^-^) (2500 in each sample) were suspended in 3 µL of 20% PBS and 80% water with 17 µL of 50 mM triethylammonium bicarbonate (TEAB) buffer, pH 8, vortexed and quickly centrifuged following sonication in ultrasound back for 10 min. Sonication of the samples was repeated for 10 min after adding 60 µL of 50 mM TEAB. Tryptic digestion was performed using 1.5 µg trypsin (sequence grade, Promega) in 50 mM TEAB buffer and incubated over night at 37°C with shaking at 600 rpm on a block heater.

TMT-10plex (Thermo Scientific) reagents in 100 µg aliquots were dissolved in 35 µL dry acetonitrile (AcN) and mixed with the digested samples (30% AcN), following incubation at 25°C for 2 h at 600 rpm. The reaction was then quenched with 12 µL of 5% hydroxylamine at 25°C for 15 min at 400 rpm. The labeled samples were pooled and dried on a speedvac (miVac). TMT-labeled tryptic peptides were then cleaned on C18 StageTips (Thermo Scientific) following solubilization with 40 µL of 5% formic acid (FA) and dried on a speedvac.

**Liquid chromatography tandem mass spectrometry**

The reconstituted samples (7 µL of 20 µL of 2% AcN/0.1% FA) were injected into a nanoLC-1000 system on-line coupled to a Q Exactive Plus mass spectrometer (Thermo Scientific, Bremen, Germany). The chromatographic separation of the peptides was achieved using a 50 cm long C18 EASY spray column (Thermo Scientific), with the following gradient: 4-26% AcN in 120 min, 26-80% AcN in 5 min and 80% AcN for 8 min at a flow rate of 300 nL/min. The MS acquisition method was comprised of one survey full scan ranging from *m/z* 350 to 1600, acquired with a resolution of R=140,000 (at *m/z* 200), followed by data-dependent HCD fragmentations from maximum 16 most intense precursor ions with a charge state 2+ and 3+. The tandem mass scans were acquired with a resolution of R=70,000, targeting 2x10^5^ ions, setting isolation width to *m/z* 2 and normalized collision energy to 33.

**Protein identification and quantification**

The raw data files were directly loaded in Proteome Discoverer v2.2 and searched against mouse SwissProt protein database (42,793 entries) using the Mascot 2.5.1 search engine (Matrix Science Ltd.). Parameters were chosen as follows: up to two missed cleavage sites for trypsin, precursor and fragment ion mass tolerance set to 10 ppm and 0.05Da, respectively. Dynamic modifications of oxidation on methionine, deamidation of asparagine and glutamine and acetylation of N-termini were used. For quantification both unique and razor peptides were requested.

**Acetoxymethyl ester Indo-1 (Indo-1-AM) Calcium Flux**

Bone marrow (BM) cells were freshly isolated and c-kit enriched using CD117 MircoBeads (Miltenyi Biotec). 2x10^6^ c-kit enriched BM cells were cultured for 30 min in complete medium supplemented with 1µM Indo-1-AM (Thermofisher Scientific) prepared as stock incubated at 37 °C for 30 min. Indo-1-AM is a cell-permeable calcium sensor dye and binds to Ca^2+^ with high affinity (Kd = 230 nM). Indo-1-AM is used to determine the changes of calcium concentrations in the cell. Subsequently the cells were washed and stained for surface markers for 15 min. Stained cells were washed and allowed to rest for 15 min in PBS with Ca^2+^. FACS tubes were run at 37°C in the sample port of the LSRII FACS equipped with a 355 nM (UV) excitation laser. Events were collected for 40 seconds prior to incubation with 1 µM human stromal cell-derived factor-1 (HuSDF-1) or murine stem cell factor (mSCF) to induce calcium transients. The average ratio, R, of bound/free Indo-1 (405 nm/485 nm emission) before simulation, was used to determine baseline values. Control samples were equilibrated in 10 mM EDTA PBS w/o Ca^2+^ to determine Rmin or stimulated with 1 µM ionomycin to determine Rmax. The following equation was used to relate Indo-1 intensity ratios to [Ca^2+^] levels and described elsewhere [7];

[Ca^2+^]=Kd*(R−Rmin)/(Rmax−R)

**Measurement of intracellular Ca^2+^ and mitochondrial Ca^2+^**

The intracellular Ca^2+^ and mitochondrial Ca^2+^ of indicated cells were determined according to the manufacturer’s instruction using Fluo-4, AM (Thermo Fisher

Scientific) and Rhod-2, AM (Thermo Fisher Scientific). In brief, 2x10^6^ c-kit enriched cells were stained with 1 μM Fluo-4, AM and 1 μM Rhod-2, AM, for 30 min at 37°C before surface staining. After staining, each fluorescent intensity was determined using a FACS. Binding of Ca^2+^ to Fluo-4 and Rhod-2 increases its fluorescence;

Fluo-4 and Rhod-2 fluorescences are calculated as 100*(Fmax-Fmin)/Fmin, where F is the measured fluorescence, and Fmin is the fluorescence before application of stimulus mSCF [6].

**Rescue experiment with SC79**

The small molecule activator of AKT (referred to as SC79) was purchased from Abcam (ab146428). C-kit enriched cells were starved for at least one hour in PBS in the absence of serum. Starved cells were incubated with 2-8 μg/ml of SC79 for 30 min at room temperature. The same concentration of DMSO (vehicle, 0.01%) was administered to the control groups.

**Extracellular flux analysis**

The oxygen consumption rate (OCR) was measured using the XFe-96 Extracellular Flux Analyzer (Seahorse Bioscience, Agilent). LSK cells (20,000-80,000) from WT and KO mice were FACS sorted and counted, resuspended in 180 µL XF assay media (non-buffered DMEM containing 10 mM glucose, 2 mM Glutamax and 1mM sodium pyruvate) and plated onto Cell-Tak-coated plates. OCR was monitored in basal conditions and in response to oligomycin (4 µM), FCCP (2 µM), rotenone (1 µM) and antimycin A (40 µM) and the values were normalized to cell number. The levels of basal respiration (**OCR_basal_–OCR_Rotenone/AntimycinA_**), ATP production (**OCR_basal_–OCR_Oligomycin_**), maximal respiration (**OCR_FCCP_–OCR_Rotenone/AntimycinA_**) and spare respiratory capacity (**OCR_FCCP_–OCR_basal_**) were calculated for each mouse and the mean ± SD values for replicates were indicated on the bar graph.

**Flow cytometry analysis and cell sorting**

Flow cytometric analyses were performed on FACSCalibur^TM^ and FACS LSRII^TM^ (BD) and FACS Sorting was performed on Aria™ (BD) at the Lund University FACS core facility. All collected data was analyzed using the FlowJo software (Tree Star).

**5-Fluorouracil treatment**

A stock solution of 5-FU (Sigma, St Louis, MO, USA) was prepared in physiologic saline at a concentration of 10 mg/ml. Each treated mouse received 1.5 mg of 5-FU per 10 g body weight via a tail vein. Marrow was removed from the treated mice 12 days later.

**Apoptotic assay**

To determine the apoptotic status of HSPC, freshly isolated and washed bone marrow cells were stained for LSKCD150^+^CD48^-^Flt3^-^CD34^-^, Annexin V-PE (559763, BD Biosciences) and DAPI. The stained samples were analyzed using FACSCalibur^TM^ (BD Bioscience).

**Cell cycle assay**

Freshly harvested bone marrow cells were fixed with BD Fix/Perm solution and incubated for 20 min at 4°C, then permeabilized with 0.25% TritonX-100 for 30 min at 4°C. Washed cells were stained with proliferation marker Ki67 and the DAPI viability staining solution and analyzed using FACS LSRII (BD Bioscience).

**Quantitative RT-PCR**

RNA from c-kit enriched or sorted 1000 LSKCD150^+^CD48^-^Flt3^-^CD34^-^ of S100A6 WT and KO cells were isolated and reverse transcribed. qRT-PCR was performed with gene specific primers, in triplicate and data were normalized with housekeeping genes ActB, GAPDH and HPRT.

***In Vitro* Culture Colony Forming Unit (CFU) Assay and Neutralizing assay**

For analysis of CFU, BM was seeded in methylcellulose (M3434, Stem Cell Technologies) at 30 000 cells/ml in 35 mm Petri dishes, according to manufacturer’s protocol. Cells were counted after 7 days of culture. For neutralizing assay, 500 freshly isolated LT-HSCs (LSKCD150^+^CD48^-^Flt3^-^CD34^-^ ) of WT cells were plated in methylcellulose (M3434, Stem Cell Technologies). WT cells were treated with or without calcyclin antibody (H-55, Santa Cruz), cultured and counted after 11 days of culture.

**Western Blot**

Sorted 20 000-50 000 LSK cells were lysed in [2x Laemmli Sample Buffer](https://www.bio-rad.com/en-se/sku/1610737-2x-laemmli-sample-buffer?ID=1610737) (Biorad) with 1% protease inhibitor cocktail and 2-Mercaptoethanol (β-mercaptoethanol) followed by centrifugation at 13 000 g for 10 min. Whole cell lysates were electrophoresed on NuPAGE™ 4 to 12%, Bis-Tris (Thermo Fisher Scientific). Proteins were transferred to nitrocellulose membranes. Membranes were then blocked with milk (2% non-fat dry milk in PBS supplemented with 0.1% Tween-20) for 1 hour and then rocked with primary antibodies overnight at 4°C and with appropriate horseradish peroxidase (HRP)-conjugated secondary antibodies for 1 hour at room temperature. Proteins were visualized by chemiluminescence detection using Amersham ^TM^ ECL ^TM^ Prime Western Blotting Detection Reagent. Quantification was performed using Image J software. T-Akt immunoblot was used as a loading control for normalization of the quantification. Anti- (Akt (pan) (11E7) Rabbit, Cell Signaling Technology), - Phospho-Akt (Ser473) (D9E) XP® Rabbit, Cell Signaling Technology), -Actin Ab-5 Purified Mouse (Becton Dickinson AB), -Rabbit IgG (H+L) Cross-Adsorbed Secondary Antibody, HRP (Thermo Fisher Scientific), -Mouse IgG (H+L) Cross-Adsorbed Secondary Antibody, HRP (Thermo Fisher Scientific).

**Statistics**

Statistical analyses were performed in Prism version 8 (GraphPad Software, San

Diego, CA, USA). Differences between groups were analyzed by an unpaired

t-test (parametric) or Wilcoxon-Mann-Whitney tests (non-parametric). For grouped analyses ANOVA were applied. Assumption of normality was tested on representative data using the D’Agostino & Pearson test, or the Shapiro-Wilk test. A description of further methods is given in the Supplementary information.

**References (Supplementary materials and methods):**

1. Kim D, Langmead B, Salzberg SL. HISAT: a fast spliced aligner with low memory requirements. Nat Methods. 2015;12:357-360.

2. Liao Y, Smyth GK, Shi W. featureCounts: an efficient general purpose program or assigning sequence reads to genomic features. Bioinformatics. 2014;30:923-930.

3. Ritchie ME, Phipson B, Wu D, Hu Y, Law CW, Shi W, et al. limma powers differential expression analyses for RNA-sequencing and microarray studies. Nucleic Acids Res. 2015;43:e47.

4. Law CW, Chen Y, Shi W, Smyth GK. voom: Precision weights unlock linear model analysis tools for RNA-seq read counts. Genome Biol. 2014;15:R29.

5. Subramanian A, Tamayo P, Mootha VK, Mukherjee S, Ebert BL, Gillette MA, et al. Gene set enrichment analysis: a knowledge-based approach for interpreting genome-wide expression profiles. Proc Natl Acad Sci U S A. 2005;102:15545-15550.

6. Drummond RM, Mix TC, Tuft RA, Walsh JV, Jr., Fay FS. Mitochondrial Ca^2+^homeostasis during Ca^2+^ influx and Ca^2+^ release in gastric myocytes from Bufo marinus. J Physiol. 2000;522 Pt 3:375-390.

7. Luchsinger LL, de Almeida MJ, Corrigan DJ, Mumau M, Snoeck HW. Mitofusin 2 maintains haematopoietic stem cells with extensive lymphoid potential. Nature. 2016;529:528-531.
